# Mental health disparities among maternal populations following heatwave exposure in North Carolina (2011–2019): a matched analysis

**DOI:** 10.1016/j.lana.2025.100998

**Published:** 2025-01-23

**Authors:** Sarah E. Ulrich, Margaret M. Sugg, Dennis Guignet, Jennifer D. Runkle

**Affiliations:** a Department of Geography and Planning, P.O. Box 32066, Appalachian State University, Boone, NC 28608, USA; b Department of Economics, P.O. Box 32051, Appalachian State University, Boone, NC 28608, USA; c North Carolina Institute for Climate Studies, North Carolina State University, 151 Patton Avenue, Asheville, NC 28801, USA

**Keywords:** Heatwaves, Excess heat factor, Matched analysis, Maternal mental health, Substance use

## Abstract

**Background:**

The increasing incidence of extreme heat due to climate change poses a significant threat to maternal mental health in the U.S. We examine the association of acute exposure to heatwaves with maternal mental health conditions in North Carolina from 2011 to 2019.

**Methods:**

We incorporate a matched analysis design using NC Hospital Discharge Data to examine emergency department admissions for psychiatric conditions during the warm season (May to September), matching heatwave periods with non-heatwave unexposed periods at the zip code tabulation area (ZCTA) level. We stratify the sample to examine effect modification across the rural-urban continuum, physiographic regions, measurements of neighborhood racial and economic inequality, and individual-level sociodemographic factors (e.g., age, race/ethnicity, and insurance type).

**Findings:**

Our sample of 324,928 emergency department visits by pregnant individuals has a mean age of 25.8 years (SD: 5.84), with 9.3% (n = 30,205) identifying as Hispanic. Relative risk (RR) estimates and 95% confidence intervals (CI) indicate significant increases in maternal mental health burdens following heatwave exposure. Acute heatwave periods were associated with a 13% higher risk of severe mental illness (RR_SMI_: 1.13, CI: 1.08–1.19, *p*: <0.0001), while prolonged exposure to moderate-intensity heatwaves was associated with 37% higher risk (RR_SMI_: 1.37, CI: 1.19–1.58, *p*: <0.001). Individual factors (e.g., advanced maternal age and insurance providers) and neighborhood-level characteristics, like low socioeconomic status, racialized and economic segregation, rurality, and physiographic region, further modified the risk of adverse maternal mental health outcomes.

**Interpretation:**

Our results add to the growing evidence of the impact of extreme heat on maternal mental health, particularly among vulnerable subpopulations. Additionally, findings emphasize the influence of socioeconomic and environmental contexts on mental health responses to heatwave exposure.

**Funding:**

This work was supported by the Faculty Early Career Development Program (CAREER) award (grant #2044839) from the 10.13039/100000001National Science Foundation and the 10.13039/100000066National Institute of Environmental Health Sciences (NIEHS) award (grant #5R03ES035170-02).

**Research in context**

**Evidence before this study**

The frequency, duration, and intensity of heatwave events are projected to increase due to climate change. However, limited research has explored the impact of heatwave exposure on maternal mental health or identified specific individual and regional risk factors that may exacerbate this risk. To address this gap, we conducted a scoping search of PubMed and Google Scholar in August 2023, using search terms such as “maternal mental health,” “heatwave exposure,” “maternal health outcomes,” “extreme heat exposure,” and “perinatal mental health.” Supplemental searches included internet searches, reference lists from identified papers, and the authors’ expertise. Studies published before April 1, 2024, were included without language restrictions.

Our research identified only two relevant studies—one from the U.S. and one from Australia—that examined the association between extreme heat exposure and maternal mental health. Both studies provided evidence suggesting that exposure to extreme heat worsens maternal mental health outcomes. However, no studies specifically examined heatwave event exposure or its direct effects on maternal health outcomes. The scarcity of prior research highlights the urgent need for further investigation into this critical area, particularly as the frequency and intensity of heatwaves increase with climate change to help inform future maternal healthcare policy and action.

**Added value of this study**

This study analyzes a large dataset of individual-level emergency department visits (n = 328,924) in North Carolina, USA, to investigate associations between heatwave exposure and maternal mental health conditions. Findings indicate that heatwave exposure increases the risk of severe maternal mental health conditions, with stronger associations observed for moderate-intensity heatwaves. The analysis highlights heightened risks among specific groups, including individuals of advanced maternal age, racial minorities, and those across various insurance categories. Additionally, neighborhood-level factors such as socioeconomic status, racial diversity, geographic location, and rural-urban context were examined to capture the complex and heterogeneous impacts of heatwaves on maternal mental health.

**Implications of all the available evidence**

This study contributes to an extremely limited body of knowledge by examining the relationship between heatwave event exposure and maternal mental health. Our findings suggest that heatwave exposure during pregnancy is associated with an increased risk of emergency department visits for maternal mental health conditions. Notably, heightened vulnerabilities were observed in subgroups such as individuals of advanced maternal age, racial minorities, and those with varying insurance statuses, as well as across neighborhood-level characteristics including socioeconomic status, racial diversity, rurality, and physiographic region. These results underscore the need for further research to explore the effects of heatwave intensity and regional susceptibility.

Additionally, the study highlights variations in mental health burdens across physiographic and rural-urban regions, stressing the importance of targeted public health interventions that may include the development of early warning systems for pregnant individuals, increased access to cooling resources, and the implementation of community-based support programs during heatwave events. Future research should focus on assessing the effectiveness of these interventions, exploring the mechanisms linking heatwaves to maternal mental health, and examining the broader impacts of climate change on maternal and child health.

## Introduction

The anticipated rise in the frequency and intensity of extreme heat events driven by climate change is expected to accelerate the decline of perinatal health in the U.S., highlighting the urgent need for research on the implications of maternal mental health in a warming climate.^1^ Maternal mental health conditions are a significant concern in the U.S., where undiagnosed or untreated cases are the leading cause of preventable pregnancy-related deaths.^2^, ^3^, ^4^ These conditions also significantly contribute to maternal and infant morbidity.^5^^,^^6^ Potential mechanisms through which extreme heat exposure can exacerbate general mental health conditions include sleep disruption, which is known to affect cognitive functioning; impaired thermoregulation, which can strain the body's ability to maintain homeostasis; and interference with medications, particularly those used to treat psychiatric conditions (e.g., antipsychotics) which can impair the body's ability to dissipate heat.^7^ While the specific biological mechanisms by which heat exposure exacerbates mental health outcomes in pregnant populations are still unclear, it is well established that pregnant people are more susceptible to increasing ambient temperatures and heatwaves due to a compromised ability to thermoregulate.^8^^,^^9^ Additionally, the adverse impacts of extreme heat exposure on birth outcomes, such as preterm birth, are well documented.^8^^,^^10^, ^11^, ^12^, ^13^, ^14^, ^15^

Although the risks of extreme heat exposure to general mental health—such as increased rates of depression, anxiety, PTSD, suicide, and psychiatric hospital admissions^16^—are well-documented, impacts on maternal mental health remain understudied. A few studies highlight this link, including one cohort study in China that found extreme heat may intensify maternal emotional and life-event stress on the day of exposure and up to two days afterward.^9^ Additionally, research in North Carolina has identified associations between short-term high ambient temperatures and an increased risk of emergency department visits for maternal psychiatric outcomes.^17^ However, no studies have assessed maternal mental health conditions following exposure to heatwave days. The objective of this analysis is to examine the association of acute exposure to heatwaves with emergency department visits for maternal mental health conditions in North Carolina from May to September 2011 to 2019. A secondary aim is to examine the moderating effects of individual- (e.g., age, race/ethnicity, insurance payer) and neighborhood-level factors (e.g., rurality and neighborhood inequality). Unlike the majority of previous work conducted at the county scale, this analysis investigates heatwave risk at a more localized sub-county scale (i.e., Zip Code Tabulation Area or ZCTA).

## Methods

### Study area

North Carolina is a state in the southeastern U.S. that faces significant challenges and disparities in maternal healthcare access and ranks poorly for maternal health outcomes compared to the rest of the nation.^18^ Heat vulnerability is shaped by a complex interaction of individual factors (e.g., age, socioeconomic status, and race/ethnicity), contextual neighborhood factors (e.g., economic status, demographic status, and rurality), and physiographic region (e.g., mountains, coast, or Piedmont).^7^ The physiographic regions of North Carolina are mapped in Supplemental Fig. S1. These factors can differ between closely situated neighborhoods,^19^ necessitating the investigation of spatial differences in maternal heatwave exposure at a finer scale (e.g., ZCTA). North Carolina's mix of urban, suburban, and rural areas presents considerable variation in socioeconomic and demographic characteristics, creating a complex landscape for studying the effects of increased maternal heatwave exposure across geographic regions and maternal subpopulations.

### Maternal mental health data

Maternal mental health data were derived from emergency department visits in North Carolina during the warm months (May to September) of 2011–2019 (Table 1). Emergency department visitation data is from the University of North Carolina’s Cecil G. Sheps Center for Health Services Research. We initially identified emergency department visits related to pregnancy complications, live births, spontaneous abortions, and elective abortions. Emergency department visits for live births, spontaneous abortions, and elective abortions were subsequently excluded, leaving only those for pregnancy-related complications. Within this sample, we then identified pregnant individuals who were admitted to the emergency department with one of five select maternal mental health disorders: perinatal mood and anxiety disorders (PMAD), comprised of pre-existing depressive and anxiety disorders that persist during pregnancy; severe mental illness (SMI), which includes bipolar and psychotic disorders; maternal mental disorders complicating pregnancy (MDP); suicidal thoughts (SUIT); and substance misuse (SUB). PMAD and SMI are pre-existing mental health conditions that recur during pregnancy, while MDP includes mental health conditions that first occur during pregnancy. Maternal mental health conditions presented to the emergency department reflect the most severe of the disease spectrum.^16^ We defined maternal mental health outcomes using either the primary or the secondary International Classification of Diseases, 9th or 10th Revision (ICD-9 ICD-10) codes for each visit to account for inconsistencies in the use of primary and secondary codes among clinicians.^17^ The ICD-10 and ICD-9 codes used to identify the pregnant population and define each maternal mental health outcome are listed in Supplemental Table S1.

**Table 1:** Summary statistics for emergency department ED visits for maternal mental health conditions for May to September of 2011–2019 in North Carolina.

Category	Strata	All Visits (n = 324,928)		PMAD (n = 2875)			MDP (n = 3808)			SMI (n = 503)			SUIT (n = 490)			SUB (n = 2894)		
		n	%	n	%	*p*^a^	n	%	*p*^a^	n	%	*p*^a^	n	%	*p*^a^	n	%	*p*^a^
Age (Years)	18–19	32,877	10.1	284	9.9	0.00039	350	9.2	0.00040	46	9.1	<0.0001	52	10.6	0.094	156	5.4	<0.0001
	20–24	120,65	37.1	995	34		1308	34.3		138	27.4		171	34.9		887	30.6	
	25–29	93,254	28.7	808	28.1		1151	30.2		188	37.4		140	28.6		979	33.8	
	30–34	50,693	15.6	500	17.4		656	17.2		74	14.7		76	15.5		599	20.7	
	35–39	21,838	6.7	218	7.6		279	7.3		50	9.9		34	6.9		213	7.4	
	40+	5613	1.7	70	2.4		64	1.7		7	1.4		17	3.5		60	2.1	
	Unknown	0	0	0	0		0	0		0	0		0	0		0	0	
Race	White	136,692	42.1	1564	54.4	<0.0001	2138	56.1	<0.0001	219	43.5	0.0085	247	50.4	0.0016	1895	65.5	<0.0001
	Black	144,428	44.4	1020	35.5		1325	34.8		242	48.1		192	39.2		819	28.3	
	Other	39,387	12.1	252	8.8		307	8.1		37	7.4		44	9		159	5.5	
	Unknown	4421	1.4	39	1.4		38	1		5	1		7	1.4		21	0.7	
Ethnicity	Not Hispanic	287,633	88.5	2601	90.5	<0.0001	3496	91.8	<0.0001	466	92.6	0.00040	440	89.8	0.11	2785	96.2	<0.0001
	Hispanic	30,205	9.3	199	6.9		210	5.5		22	4.4		35	7.1		63	2.2	
	Unknown	7090	2.2	75	2.6		102	2.7		15	3		15	3.1		46	1.6	
Insurance	Self-Pay	55,438	17.1	343	12	<0.0001	454	12	<0.0001	58	11.7	<0.0001	51	10.4	<0.0001	656	22.7	<0.0001
	Medicaid	179,627	55.4	1608	56.1		2291	60.4		297	59.8		300	61.2		1758	60.8	
	Private	74,065	22.8	712	24.8		746	19.7		67	13.5		95	19.4		355	12.3	
	Other Gov't	9573	3	92	3.2		137	3.6		13	2.6		18	3.7		39	1.3	
	Other	4899	1.5	104	3.8		156	4.1		56	11.4		26	5.3		80	2.8	
	Unknown	663	0.2	8	0.1		12	0.3		6	1.1		0	0		3	0.1	
RUCA	Rural	18,107	7	201	8.7	0.0024	186	6.2	0.0093	23	5.5	0.12	18	4.6	0.053	217	8.3	0.00043
	Suburban	45,159	17.4	385	16.7		468	15.7		93	22.4		67	17.2		494	19	
	Urban	196,731	75.7	1715	74.5		2330	78.1		300	72.1		305	78.2		1893	72.7	
Region	Coastal	85,425	32.9	793	34.5	0.0095	1017	34.1	0.0028	150	36.1	0.83	146	37.4	0.15	802	30.8	<0.0001
	Piedmont	141,441	54.4	1197	52		1553	52		220	52.9		189	48.5		1332	51.2	
	Western	33,131	12.7	311	13.5		414	13.9		46	11.1		55	14.1		470	18	
ICE Income	Q1	49,600	15.3	413	15.9	0.34	554	14.7	0.032	76	16.2	0.50	71	16.7	0.63	553	17.7	<0.0001
	Q2	54,824	16.9	448	17.3		568	15.1		77	16.5		80	18.8		614	19.7	
	Q3	69,787	21.5	572	22.1		844	22.4		102	21.8		89	20.9		779	25	
	Q4	88,269	27.2	687	26.5		1007	26.8		124	28.6		120	28.2		778	24.9	
	Q5	61,630	19	470	18.1		789	21		79	16.9		66	15.5		396	12.7	
ICE Race	Q1	116,198	35.8	824	31.8	0.0011	1115	29.6	<0.0001	171	36.5	0.38	163	38.3	0.64	927	29.7	<0.0001
	Q2	79,881	24.6	640	24.7		919	24.4		127	27.1		100	23.5		734	23.5	
	Q3	72,302	22.3	620	23.9		925	24.6		96	20.5		86	20.2		679	21.8	
	Q4	41,783	12.9	371	14.3		593	15.8		58	12.4		63	14.8		520	16.7	
	Q5	14,021	4.3	136	5.2		213	5.7		16	3.4		14	3.3		260	8.3	

PMAD = Perinatal mood and anxiety disorders; MDP = Maternal mental disorders of pregnancy; SMI = Severe mental illness; SUI-T = Suicidal thoughts; SUB = Substance misuse; RR = Relative risk; CI = Confidence interval; RUCA = Rural-Urban Commuting Area Codes; ICE = Index of Concentration of Extremes.

a *p*-values from Chi-square test results for differences in proportions across categorical groups.

We obtained individual demographic data from each emergency department record, including patient race (American Indian, Asian, Black, Native Hawaiian or Pacific Islander, White, and Other), ethnicity (Hispanic or Non-Hispanic), payer source (i.e., Medicaid, private or commercial insurance, self-pay/uninsured, and other), and age in years. Race categories were grouped into Black, White, and Other. Data with missing or unknown values for race, age, ethnicity, or insurance (as outlined in Table 1) were excluded from the analysis. Missing data accounted for less than 2% of the total dataset.

### Heatwave calculation

We identified heatwave days using the Excess Heat Factor (EHF), a metric that accounts for local temperature extremes relative to an area’s typical climate.^20^ As there is no universally accepted definition for heatwaves nor a well-established critical period of maternal sensitivity to high temperatures,^10^^,^^15^^,^^21^ it is difficult to define the thresholds for and intensity of heatwave risks.^22^ Excess Heat Factor is a valuable approach for assessing heatwaves across North Carolina, a state with a diverse geography and climate across the western mountainous, central Piedmont, and eastern coastal regions. In contrast to using a uniform absolute temperature threshold statewide, the Excess Heat Factor adjusts for geographic variations in heat tolerance by identifying the 95th percentile threshold specific to each ZCTA location over the study period, and then identifying temperatures in excess of the 95th percentile value over three or more days relative to a preceding 30-day acclimatization period.^22^ This acclimatization period accounts for physiological processes influencing individual and population responses to heatwaves.^22^ The choice of three days in Excess Heat Factor calculations is supported by studies of human physiological responses to extreme heat.^20^ Following methodology outlined by Nairn and Fawcett, we used daily average ambient temperatures at the ZCTA level to calculate the Excess Heat Factor.^20^ This finer-scale approach at the ZCTA level considers local variations in heat exposure. Excess Heat Factor categorizes heatwaves into different intensity levels: low (EHF < 1 and > 0), moderate (EHF≥1 and < 2), and high intensity (EHF > 2). In our primary analysis, we included all heatwave intensities and stratified heatwave days by intensity level in our sub-analyses.

The use of different heatwave definitions can change the days identified as heatwaves. To assess the sensitivity of our results to alternative heatwave definitions, we estimated the risk of emergency department visits for maternal mental health outcomes using two alternative definitions. Alternative heatwave definitions were based on three or more consecutive days above a 95th percentile average ambient temperature or heat index threshold based on 1) daily average temperature or 2) daily heat index at the ZCTA level from May to September over the entire 2011 to 2019 period (i.e., warm season). Daily heat index values during the study period were calculated using daily average temperature and relative humidity at the ZCTA level using the “weathermetrics” package in RStudio version 2023.12.0 and R version 4.3.1.

### Exposure periods

Prior research demonstrates increased mental health risks from heatwaves on the same day of exposure (lag0)^23^ and delayed risks up to 3–6 days following exposure.^24^, ^25^, ^26^ We evaluated the effect of heatwave exposure on single-day lags up to seven days following a heatwave day and across cumulative lag periods. Evaluating exposure using cumulative lags over heatwave periods captures the cumulative effects of heat over time. This approach considers how health effects can emerge after prolonged exposure to high temperatures, reflecting maternal stress and physiological strain accumulation over an extended period.^17^

We evaluated the cumulative impact of acute and prolonged heatwave exposure on the incidence of maternal mental health-related emergency department visits. Acute heatwave periods were defined as a heatwave day on the day of admission and the three days immediately following it (lag0 to lag3), and prolonged heatwave periods encompassed a heatwave day on the day of admission and the subsequent seven days (lag0 to lag7). We also examined the effect of single-day lags (heatwave day on the day of the emergency department visit and up to seven days following exposure).

### Covariates

Using demographic data from individual-level emergency department records, we investigated maternal age, race/ethnicity, and socioeconomic status as individual effect modifiers.^12^^,^^27^, ^28^, ^29^, ^30^, ^31^ Previous studies highlighted elevated mental health burdens in Black, Hispanic, and low-income maternal populations in North Carolina, particularly during periods of extreme temperature (Runkle et al., 2024). We assessed socioeconomic status using insurance type (i.e., Medicaid enrollment) (Qu et al., 2021). Individuals were categorized based on advanced maternal age (≥35 or <35), race (Black, White, or Other), insurance type (Private, Medicaid, Self-Pay, Other), and ethnicity (Hispanic or Non-Hispanic). Due to smaller sample sizes, American Indian, Asian, and Native Hawaiian or Pacific Islander groups were aggregated into an “Other race” category. Women aged ≥35 were classified as “advanced maternal age” per American Medical Association guidelines, reflecting established clinical thresholds for heightened risks and aligning with standard risk stratification practices. This categorization ensures clinical relevance, as emphasized by Binney et al. (2023), who supports its utility in translating research findings into practice. To evaluate the robustness of our findings to variations in age categorization, we conducted a sensitivity analysis using alternative age groupings: 18–24, 20–24, 25–29, 30–34, 35–39, and ≥40 years.

We considered the rural-urban status and measurements of economic and racial inequality as ZCTA-level structural factors that may have modified the effect of extreme heat exposure on maternal mental health outcomes. Rural-Urban Commuting Area (RUCA) codes from the USDA Economic Research Service were used to classify each ZCTA as Rural (1–3), Suburban (4–6), and Urban (7–10).^17^^,^^32^, ^33^, ^34^ RUCA scores were mapped in Supplemental Fig. S2.

Residential segregation, a proxy for structural racism, and neighborhood economic inequality have previously been associated with poor mental health during pregnancy, as well as adverse maternal health effects following heatwave exposure.^17^^,^^35^^,^^36^ Structural racism at the neighborhood level creates disparities in healthcare access and healthcare barriers that have been shown to contribute to additional psychological stressors and exacerbate mental health outcomes for Black women.^37^, ^38^, ^39^ We utilized the Index of Concentration at the Extremes (ICE) to assess the impact of structural racism and economic polarization on maternal mental health outcomes.^40^, ^41^, ^42^, ^43^, ^44^, ^45^ ICE scores range from −1 to 1 and can indicate the degree of concentration of racial (or economic) extremes in each ZCTA. Negative scores signify a high concentration of minority (or low-income) populations, while positive scores indicate a concentration of white (or affluent) residents. ICE metrics were calculated using data from the American Community Survey 2013–2017 with 5-year estimates and divided into quintiles, mapped in Supplemental Fig. S3. Supplemental Table S2 details the methodology used to derive ICE Race and ICE Income scores.

Finally, we evaluated differences across geographic regions (i.e., Western, Piedmont, and Coastal) following previous studies assessing maternal heatwave exposure and preterm birth in North Carolina.^36^^,^^46^ Assessing heatwave exposure and mental health responses by region accounts for our study area's significant physical and climatic regional variation.

### Matched design and statistical analysis

Summary statistics and chi-square tests assessed demographic and categorical differences in the study population. Our primary analysis quantified the association between heatwave exposure and maternal mental health outcomes. Similar to other matched case design analyses,^47^, ^48^, ^49^ we also stratified our matched analyses to assess whether different individual, regional, or neighborhood characteristics were associated with elevated risks of mental health impacts following maternal exposure to extreme heat.

Following a well-established methodology for analyzing health outcomes associated with heatwaves like preterm birth,^50^ hospital admissions,^47^^,^^51^ and mortality,^52^ we matched each heatwave day to eligible non-heatwave days to compare maternal mental health outcomes within matched strata. Consistent with prior literature,^53^ each heatwave day was matched with three randomly selected non-heatwave days from the same ZCTA, occurring within two calendar days before or seven days after the heatwave day, but in a different year. Days that fell within a three-day window of a different heatwave event in the same ZCTA were excluded from being selected as controls. Following established studies on extreme temperatures and maternal mental health outcomes, we included three- and seven-day lags to capture cumulative exposure effects.^17^ This matching procedure reduces biases due to otherwise confounding factors, including seasonal trends.^47^^,^^48^^,^^50^, ^51^, ^52^ Our final sample size of emergency department visits was determined by (1) the availability of non-heatwave control days to match with heatwave days, and (2) the number of lag days included in the analysis to define heatwave periods for each heatwave-exposed day (i.e., single, three- and seven-day lags).

After matching, we used conditional quasi-Poisson models to estimate the relative risk (RR) of maternal mental health outcomes during heatwave days and lag periods compared to matched non-exposed days, incorporating a distributed lag for heatwave exposure.^47^^,^^52^^,^^54^ For each maternal mental health condition, the dependent variable in the models was the daily count of the outcome, with observation values weighted by the maternal population in each ZCTA. Models were fitted using the gnm() function in R,^55^ with eliminate = factor(ZCTA) to control for ZCTA-level variation and a quasi-Poisson distribution with a log link function to account for overdispersed count data. The models also included adjustments for day of the week, year, and a log-transformed population offset to control for temporal and population-level confounders. Relative risks (RRs) estimates with 95% confidence intervals (CIs) were calculated for single-day lags up to seven days following heatwave days, and to assess the cumulative impact of acute (lag0 to lag3) and prolonged (lag0 to lag7) heatwave exposure.^17^ We conducted sensitivity analyses to ensure the robustness of the primary results to alternative statistical modeling assumptions. The tested alternative models included log-linear mixed-effects models, which are commonly applied in other matched designs,^48^^,^^52^ as well as generalized linear mixed models and conditional Poisson models.

Associations with the individual (e.g., age, race, ethnicity, and insurance subgroups), community-level (e.g., ICE Income, ICE Race, and RUCA subgroups), and regional factors (e.g., Western, Piedmont, and Coastal) were analyzed by using stratified samples. Separate models were estimated within each stratum, and the relative risk (RR) estimates were compared across strata to evaluate differences in the associations between heatwave exposure and each maternal mental health outcome. We assessed effect modification in the association between heatwaves and maternal health outcomes across subgroups (e.g., Age, Race, Region). The differences in estimated RR within each subgroup were evaluated using the calculated as follows:

$$W=\left({\left(\beta_{1}\;-\;\beta_{2}\right)}^{2}\right)/\left(S{E_{1}}^{2}+S{E_{2}}^{2}\right)$$

Where β_1_ and β_2_ are the estimated relative risk values (or coefficients) for two groups (e.g., Rural vs. Urban), and SE_1_ and SE_2_ are their standard errors.^56^^,^^57^ The statistic follows a Chi-squared distribution with 1 degree of freedom, and *p*-values < 0.05 were considered significant, indicating potential effect modification.

All analyses were conducted in RStudio version 2023.12.0 and R version 4.3.1. with the “dlnm” package for the distributed lag non-linear models, the “gnm” package for generalized nonlinear and conditional Poisson models, the “glm” package for generalized linear mixed models, the “lme4” package for log-linear mixed-effects models, and the “forestploter” package for forest plots of the results. This study adhered to the Strengthening the Reporting of Observational Studies in Epidemiology (STROBE) guidelines for observational research (see Supplemental Material).

### Role of the funding source

The funding source of this study was not involved in its design, data collection, analysis, interpretation, or writing.

## Results

**Fig. 1:**
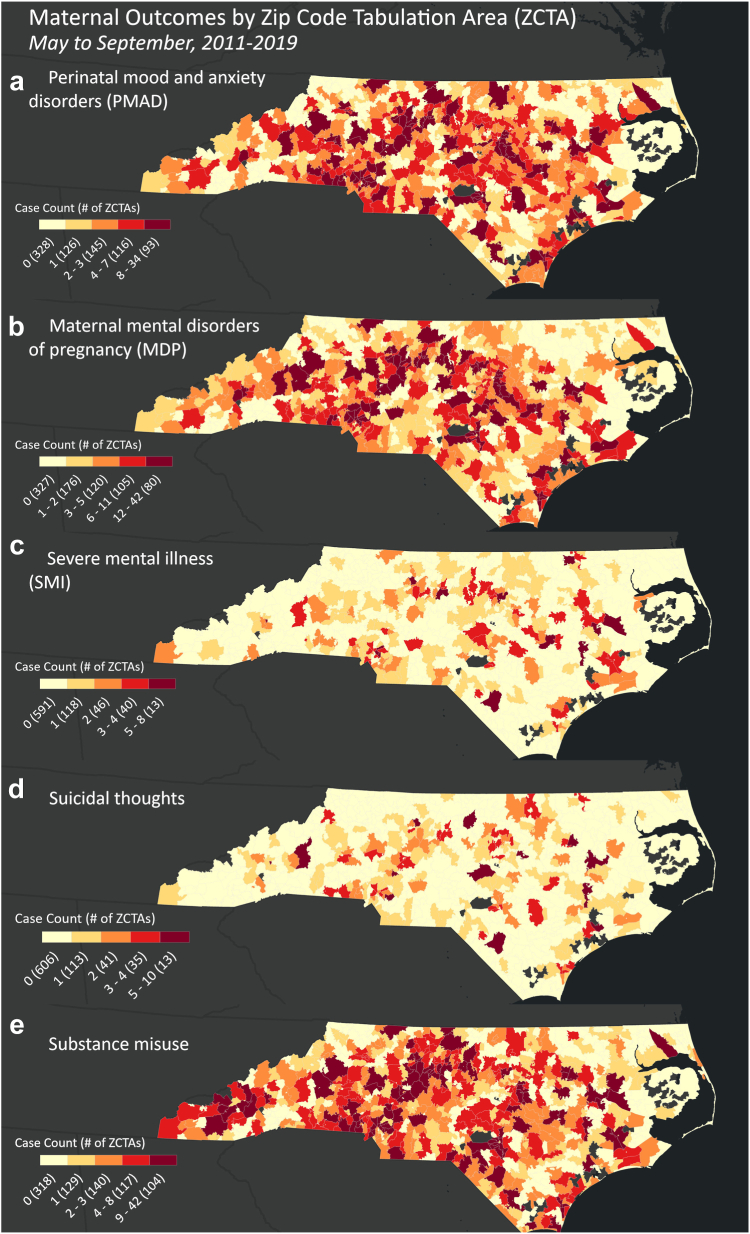
Numbers of emergency department visits for each maternal mental health outcome from May to September of 2011 to 2019, aggregated to the zip code tabulation area (ZCTA) level.

Fig. 1 shows the geographic distribution of emergency department visits for each maternal mental health condition at the ZCTA level across North Carolina. The socio-demographic distribution of the sample and the Chi-square test results for differences in proportions of categorical variables are presented in Table 1. Of the 324,928 emergency department visits during the study period included in our sample prior to heatwave matching, the majority were from Medicaid beneficiaries (179,627; 55.4%), residents of urban zip codes (196,731; 75.7%), and individuals aged 20 to 29 (213,907; 65.8%) with the mean age being 25.8 years (SD: 5.84). Most emergency department visits (144,428; 44.4%) and the majority of SMI cases (242; 48.1%) pertained to Black birthing populations. In contrast, the majority of PMAD (1564; 54.4%), MDP (2138; 56.1%), suicidal ideation (247; 50.4%), and substance misuse (1895; 65.5%) cases were white birthing populations. 10,379 (3.1%) observations were excluded from our analysis due to missing race, ethnicity, or insurance type.

There were 218 unique heatwave days in North Carolina from 2011 to 2019. Supplemental Fig. S4 shows the total number of heatwave days at the ZCTA level (n = 804). Stratification by heatwave intensity shows that high-intensity heatwave days were more frequent in the southeastern and coastal regions of North Carolina, while low- and moderate-intensity heatwave days were more common in the eastern part of the state during the study period (Supplemental Fig. S4). On average, each ZCTA experienced 43.86 (±6.58) heatwave days over the 9-year study period, ranging from 22 to 65 days, resulting in a total of 35,365 heatwave-exposed days across all ZCTAs. Supplemental Tables S3 and S4 detail the number of heatwave days included in acute and prolonged heatwave periods, matched unexposed periods, and the counts of mental health emergency department visits for each outcome with corresponding sociodemographic variables.

### Impacts of heatwaves on maternal mental health

For single-day lags, RR and 95% CI estimates are plotted in Supplemental Fig. S5, and corresponding *p*-values are reported in Supplemental Table S5. We generally did not observe a significant relationship between heatwave exposure from lag 0 to lag 7 for PMAD, MDP, substance misuse (SUB), or suicidal ideation (SUIT), with the exception of lag 7 (RR_SUIT_: 1.28, CI: 1.07–1.54, *p* = 0.0069). However, SMI risk was elevated on lag 4 (RR_SMI_: 1.46, CI: 1.21–1.76, *p* < 0.0001) and lag 6 (RR_SMI_: 1.34, CI: 1.11–1.63, *p* = 0.0028). Interestingly, we observed a protective effect on heatwave days for suicidal ideation at lag 3 (RR_SUIT_: 0.78, CI: 0.63–0.98, *p*: 0.030), for substance misuse at lag 5 (RR_SUB_: 0.83, CI: 0.73–0.96, *p*: 0.01), and for SMI at lag 7 (RR_SMI_: 0.80, CI: 0.67–0.96, *p*: 0.015) (Supplemental Fig. S5 and Table S5).

**Fig. 2:**
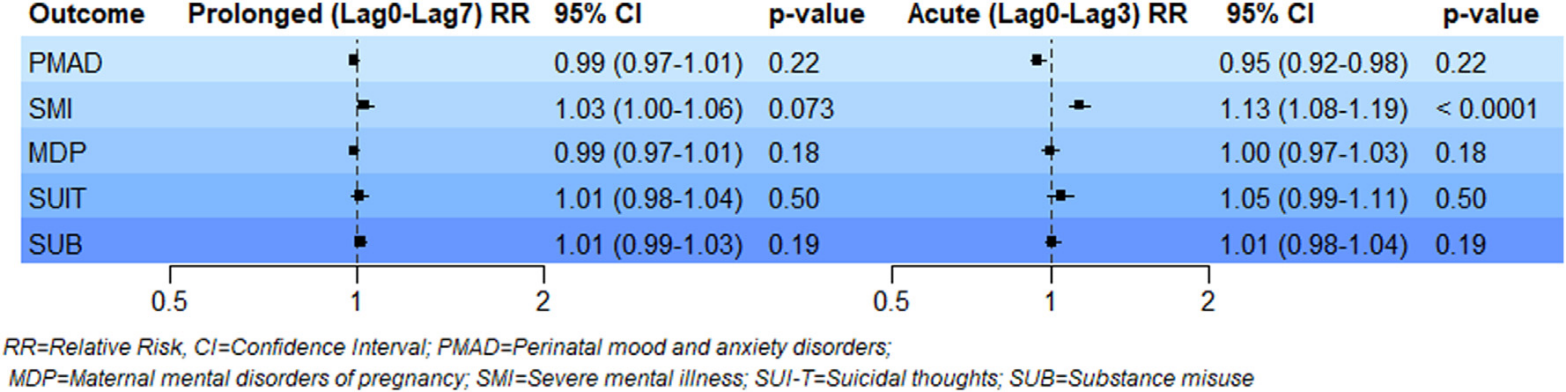
Relative risk (RR), 95% confidence intervals (CIs), and *p*-values for maternal mental health emergency department visits during acute and prolonged heatwave periods.

We identified an elevated risk of emergency department visits for SMI during acute heatwave exposure periods (lag0-lag3) (RR_SMI_: 1.13, CI: 1.08–1.19, *p*: <0.0001) and marginally significant risk for prolonged heatwave exposure periods (lag0-lag7) (RR_SMI_: 1.03, CI: 1.00–1.06, *p*: 0.073) (Fig. 2). For MDP, suicidal ideation (SUIT), substance misuse (SUB), and PMAD, we generally observed null results during both acute (lag0–lag3) and prolonged (lag0–lag7) exposure periods. However, acute exposure did reveal an insignificant protective effect for PMAD (RR_PMAD_: 0.95, CI: 0.92–0.98, *p*: 0.22).

**Fig. 3:**
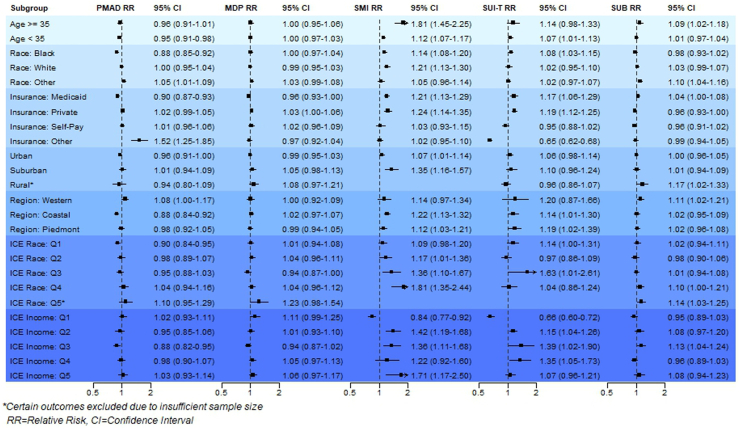
Relative risk (RR), 95% confidence intervals (CIs), and *p*-values for maternal mental health emergency department visits during acute (Lag0-Lag3) heatwave periods, stratified by subgroup.

Fig. 3 presents stratified model results for acute heat wave periods across all subgroups. The corresponding *p*-values and *p*-values for effect modification (*p-*EM) are provided in Supplemental Tables S6 and S7, respectively. Effect modification was evident for advanced maternal age and insurance type. Pregnant individuals aged 35+ demonstrated an elevated risk of emergency department visits for SMI (RR_SMI_: 1.81, CI: 1.45–2.25, *p* < 0.0001, *p*-EM: <0.0001), while individuals with ‘Other’ insurance had an increased risk for PMAD (RR_PMAD_: 1.52, CI: 1.25–1.85, *p* < 0.0001, *p*-EM: <0.0001), and individuals with Medicaid had an increased risk for substance misuse (RR_SUB_: 1.04, CI: 1.00–1.08, *p*: 0.030, *p*-EM: 0.0044) during acute heat wave periods. Individuals with “Other Race” also had effect modification with substance misuse (RR_SUB_: 1.10, CI: 1.04–1.16, *p:* 0.0007; *p*-EM: 0.05). Among neighborhood-level characteristics, risk estimates were elevated across rural-urban and geographic regions for select maternal mental health outcomes. In contrast, protective associations were observed for Black race (RR_PMAD_: 0.88, CI: 0.85–0.92, *p* < 0.0001, *p*-EM <0.0001), mixed-income neighborhoods (ICE Income Q3 RR_PMAD_: 0.88, CI: 0.82–0.95, *p*: 0.0008, *p*-EM: 0.020), low-income neighborhoods (ICE Income Q1 RR_SMI_: 0.84, CI: 0.77–0.92, *p*: 0.0002, *p*-EM: 0.00035; RR_SUIT_: 0.66, CI: 0.60–0.72, *p*-value: <0.0001, *p*-EM < 0.0001), neighborhood racial segregation (ICE Race Q1 RR_PMAD_:0.90, CI: 0.84–0.95, *p*-value: 0.0003, *p*-EM: 0.013), Medicaid enrollment (RR_PMAD_: 0.90, CI: 0.87–0.93, *p* < 0.0001, *p-*EM < 0.0001); and ‘Other’ insurance types (RR_SUIT_: 0.65, CI: 0.62–0.68, *p* < 0.0001, *p*-EM <0.0001).

**Fig. 4:**
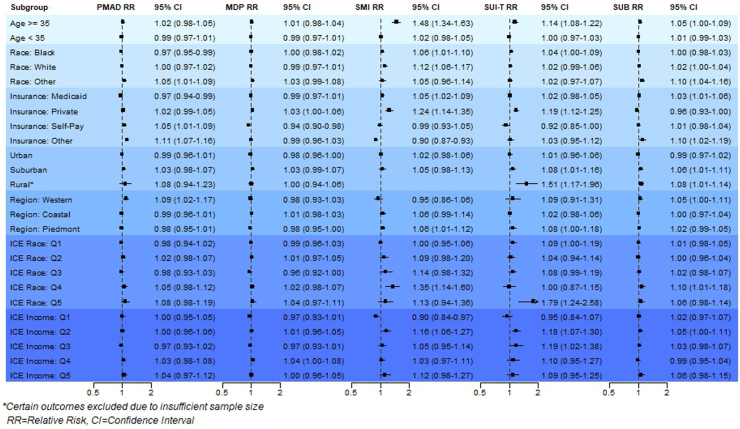
Relative risk (RR), 95% confidence intervals (CIs), and *p*-values for maternal mental health emergency department visits during prolonged (Lag0-Lag7) heatwave periods, stratified by subgroup.

Fig. 4 presents stratified model results for prolonged heatwave periods across all subgroups, with corresponding *p-* and *p-*EM values in Supplemental Tables S8 and S9, respectively. Similar to acute heatwave periods, advanced maternal age emerged as a key effect modifier (RR_SMI_: 1.48, CI: 1.34–1.63, *p* < 0.0001, *p*-EM: <0.0001; RR_SUIT_: 1.14, CI: 1.08–1.22, *p* < 0.0001*, p*-EM: 0.00011), in addition to residence in Western (RR_PMAD_: 1.09, CI: 1.02–1.17, *p*: 0.0086, *p*-EM: 0.0039) or rural (RR_SUIT_: 1.51, CI: 1.17–1.96, *p*-value: 0.0015; RR_SUB_: 1.08; CI: 1.01–1.14, *p*: 0.015, *p-*EM: 0.016) regions. Additional effect modification was observed across insurance categories, neighborhood residential segregation (e.g., ICE Race), and neighborhood economic segregation (e.g., ICE Income). Protective effects were noted for low-income neighborhoods (ICE Income Q1 RR_SMI_: 0.90; CI: 0.84–0.97, *p*: 0.0048, *p*-EM: 0.0044) and ‘Other’ insurance type (RR_SMI_: 0.90; CI: 0.87–0.93, *p* < 0.0001, *p*-EM < 0.0001).

### Sensitivity analyses

Supplemental Figs. S6 and S7 provide RR and 95% CI estimates for heatwave intensities and alternative heatwave definitions, respectively. Corresponding *p*-values are reported in Supplemental Tables S10 and S11. Acute exposure to moderate-intensity heatwaves associated with an increased risk for SMI (RR_SMI_: 1.56; CI: 1.32–1.85, *p*: <0.0001), MDP (RR_MDP_: 1.17; CI: 1.04–1.32, *p*: 0.0096), and suicidal ideation (RR_SUIT_: 1.55; CI: 1.30–1.86, *p*: <0.0001). Prolonged moderate-intensity heatwaves were associated with an elevated risk for SMI (RR_SMI_: 1.37; CI: 1.19–1.58, *p*: <0.0001) and MDP (RR_MDP_: 1.10; CI: 1.01–1.20, *p*: 0.035) but showed protective effects for PMAD (RR_PMAD_: 0.85; CI: 0.77–0.93, *p*: 0.00030) and suicidal ideation (RR_SUIT_: 0.33; CI: 0.28–0.38, *p*: <0.0001). Low-intensity heatwaves displayed mixed results, with some increased risks for SMI (RR_SMI_: 1.11; CI: 1.01–1.20, *p*: 0.011) and reduced risks for other outcomes, including suicidal ideation (RR_SUIT_: 0.81; CI: 0.74–0.90, *p*: <0.0001) and substance misuse (RR_SUB_: 0.94; CI: 0.89–0.98, *p*: 0.0073). Alternative heatwave definitions corroborated the main findings, demonstrating elevated risks for suicidal ideation and SMI during 95th percentile ambient temperature (RR_SUIT_: 1.14, CI: 1.08–1.21, *p*: 0.38; RR_SMI_: 1.08, CI: 1.01–1.17, *p*: 0.035) and heat index thresholds (RR_SUIT_: 1.21, CI: 1.05–1.39, *p*: 0.010). Protective effects (RR_SUIT_: 0.82, CI: 0.71–0.93, *p*: 0.0026) and for MDP (RR_MDP_: 0.87, CI: 0.81–0.94, *p*: 0.00040) were observed for MDP and suicidal ideation during prolonged heat index heatwave periods.

Supplemental Figs. S8 and S9 plot RR and 95% CI estimates for additional age groups during acute and prolonged heatwave periods, with corresponding *p*-values reported in Supplemental Tables S12 and S13. Younger age groups (18–24 and 25–29) exhibited increased risks during both acute (18–24 RR_SMI_: 1.27, CI: 1.17–1.39, *p* < 0.0001; 18–24 RR_SUIT_: 1.09, CI: 1.01–1.18, *p*: 0.033; 25–29 RR_SUIT_: 1.22, CI: 1.07–1.38, *p*: 0.0023) and prolonged heatwaves (25–29 RR_SUB_: 1.04, CI: 1.01–1.07, *p*: 0.010; 25–29 RR_SMI_: 1.11, CI: 1.05–1.16, *p* < 0.0001), though some protective effects were noted during acute periods (18–24 RR_PMAD_: 0.95, CI: 0.92–0.99, *p*: 0.012; 25–29 RR_PMAD_: 0.94, CI: 0.90–0.98, *p*: 0.0073; 25–29 RR_SUB_: 0.94, CI: 0.91–0.98, *p*: 0.0041). For individuals aged 30–34, heightened risks were observed during both acute (RR_SUIT_: 1.43, CI: 1.24–1.64, *p* < 0.0001) and prolonged periods (RR_SMI_: 1.22, CI: 1.14–1.31, *p* < 0.0001). Older age groups (35–39 and 40+) demonstrated higher risks for substance misuse and SMI during acute (e.g., 40+ RR_SUB_: 1.75, CI: 1.39–2.20, *p* < 0.0001) and prolonged events (e.g., 40+ RR_SMI_: 1.37, CI: 1.26–1.49, *p* < 0.0001).

## Discussion

Our study, using a robust matched design analysis, demonstrated an association between exposure to heatwaves and increased maternal emergency department visits for mental health conditions. Notably, we observed a lagged effect of heatwave exposure on the risk of emergency department visits for SMI, suggesting delayed impacts on severe mental health outcomes. Acute heatwave periods of exposure were also linked to increased SMI risk, and moderate-intensity heatwaves showed the strongest association with elevated emergency department risks. This pattern may suggest increased adaptation during high-intensity heat periods that mitigate the impacts of extreme heat on maternal mental health. Additionally, protective effects on specific lag days and among certain subgroups highlight potential resilience or adaptive responses following heat exposure. Higher emergency department visit risks were evident among pregnant individuals of advanced maternal age and across insurance groups. Neighborhood-level characteristics, including socioeconomic status, racialized and economic segregation, geographic location, and rural-urban context, also shaped mental health responses to heat exposure. These findings highlight the complex and heterogeneous impacts of heatwaves on maternal mental health, varying across time lags, demographic subgroups, and environmental contexts. Black maternal populations were overrepresented in our sample, suggesting disproportionate mental health burdens faced by this group.

Prior research has examined elevated ambient temperature associated with maternal mental health outcomes.^17^ However, our study focuses specifically on heatwave events, which consist of several consecutive days of elevated temperature above a certain threshold. These sustained periods of heat may pose cumulative or prolonged risks that differ from those associated with isolated extreme heat days. Our matched design may be more effective than other designs for heatwave analysis as it allows for the analysis of multi-year, multi-site studies, offering (1) more precise and less biased estimates of exposure-health risk associations, (2) evidence of consistency across multiple exposure events, and (3) a clearer understanding of heterogeneity and its contributing factors across different events and locations. This research, drawing on data from over 300,000 maternal emergency department visits, adds to an emerging evidence base on the impact of heatwaves on maternal mental health, a key contributor to maternal mortality in the U.S.^58^

Elevated risks for SMI were observed during acute heatwave periods (lag0-3) compared to non-heatwave periods. The association between periods of acute exposure (i.e., three to four days following a heat event or elevated ambient temperature) to extreme heat and increased hospital admissions is well-documented in the general population for psychiatric conditions,^24^^,^^59^^,^^60^ including anxiety, depression, bipolar disorder, and schizophrenia.^25^^,^^26^^,^^61^^,^^62^ Prior research in maternal populations has also found an elevated risk of maternal emotional stress during acute lag periods (lag0-3 days) following exposure to extremely high temperatures.^9^

Maternal mental health conditions, including PMAD and MDP, have been identified as significant risk factors for severe maternal morbidity.^6^ Prior studies identified an increased risk of PMAD following high-temperature exposure,^17^ which we observed in select subgroups in our analysis (e.g., Other’ insurance, Other’ race, and Western region).

Pregnant individuals of advanced maternal age also face an elevated risk of severe maternal morbidity, to which mental health conditions contribute significantly.^63^, ^64^, ^65^ Advanced maternal age was found to increase the risk of emergency department visits following heatwave exposure to SMI and suicidal ideation, underscoring the urgent need to address the impact of climate-related stress on maternal health, particularly in individuals over the age of 35.

Previous research found a positive association between suicide risk and elevated ambient temperature.^66^ Additionally, limited evidence suggests that lower socioeconomic status is associated with a heightened risk of suicide during the peripartum period.^67^ Our finding of elevated emergency department visits for suicidal ideation among several maternal subpopulations suggests that this risk is reinforced and exacerbated by both acute and prolonged heatwave exposure.

The western mountain region of North Carolina showed heightened risk of mental health outcomes including PMAD and substance misuse following heatwave exposure, while rural areas in North Carolina exhibited elevated risks of substance misuse and suicidal ideation. Both of these regions include areas designated as maternity care deserts and characterized by unique demographic and economic profiles, and have previously been identified as areas with elevated mental health burdens.^33^^,^^68^ These results indicate that the impact of heatwave exposure on maternal mental health is intensified in these underserved areas.

Our study has several strengths, including a matched analysis method that allowed for robust comparisons of maternal mental health outcomes during heatwave periods and accounted for sub-regional acclimation at the ZCTA level. The matched analysis approach contrasts within matched strata, which eliminates confounding introduced by factors that vary across strata.^53^^,^^69^ Limitations of our study include the assumption that all residents in a ZCTA were uniformly exposed to heatwave days, without considering access to heat mitigation resources like air conditioning. The matched-case-only design limits the ability to assess effect modification through additive interaction, highlighting the need for future analysis to better understand the potential causal effects of heatwaves. Additionally, our sample did not include data on psychiatric medications, which can interact with extreme heat exposure. For this analysis, we assumed that race, age, and other data for individual characteristics in our emergency department visits were missing completely at random (MCAR). We opted to use a complete case analysis, involving only analyzing cases with no missing data on the variables of interest. This approach can reduce the overall sample size of the study population with the potential to decrease the study’s statistical power and could lead to differences in our sample compared to the broader population of maternal emergency department visits. We are restricted to categorical data for the majority of our key variables, which are reported as categorical or categorized as ordinal data. The categorization of quantitative variables in our analysis (e.g., age, ICE Income, ICE Race, and RUCA values) may result in loss of statistical power, inefficiency, and residual confounding.^70^ Studies should include maternal populations outside North Carolina and directly measure access to protective resources to confirm the generalizability of our findings.

In conclusion, this study underscores the heightened vulnerability of maternal populations to mental health risks during heatwaves events of varying intensity, particularly in the southeastern coastal region of North Carolina. Findings highlight the urgent need for targeted public interventions, such as early warning systems tailored for pregnant individuals, increased access to cooling resources, and the establishment of community-based support programs.^71^ These strategies have the potential to mitigate long-term consequences for mothers and their children, addressing a critical gap in mental health resilience in a changing climate. Future studies should prioritize evaluating the effectiveness of these interventions and advancing our understanding of the biological mechanisms linking heatwave exposure to psychiatric outcomes in maternal populations.

## Contributors

S.U.: Conceptualization, Methodology, Formal analysis, Investigation, Data curation, Writing—original draft, Writing—review & editing, Visualization.

M.S.: Conceptualization, Methodology, Formal analysis, Data curation, Writing—original draft, Writing—review & editing, Supervision, Project administration, Funding acquisition.

D.G.: Methodology, Writing—review & editing, Supervision.

J.R.: Conceptualization, Methodology, Writing—original draft, Writing—review & editing, Supervision, Project administration, Funding acquisition.

SU, MS, and JR directly accessed and verified the underlying data reported in the manuscript and are responsible for the decision to submit the manuscript.

## Data sharing statement

Emergency department data for this study were obtained from the University of North Carolina’s Cecil G. Sheps Center for Health Services Research and is not available for public sharing.

## Declaration of interests

The authors declare no competing interests.
