# Supplementary material for: Mental health disparities among maternal populations following heatwave exposure in North Carolina (2011–2019): a matched analysis
Source: Lancet Reg Health Am. 2025 Jan 23;42:100998. doi: 10.1016/j.lana.2025.100998 (PMC11804822; doi:10.1016/j.lana.2025.100998)

**Mental Health Disparities among Maternal Populations Following Heatwave Exposure in North Carolina (2011–2019): A Matched Analysis**

**Supplemental Materials**

**Table of Contents**

Supplemental Table 1……………………………………………………………………………………………...1

Supplemental Table 2…………………...…………………………………………………………………………5

Supplemental Table 3…………………………….…..……………………………………………………………6

Supplemental Table 4………………………………...….………………………………………………………...7

Supplemental Table 5……………………..….……………………………………………………………………8

Supplemental Table 6……………………………………………………………………………………………...9

Supplemental Table 7…………………...………………………………………………………………………..10

Supplemental Table 8…………………...………………………………………………………………………..11

Supplemental Table 9…………………...………………………………………………………………………..12

Supplemental Table 10………..……..…………………………………………………………………………...13

Supplemental Table 11……..………..…………………………………………………………………………...14

Supplemental Table 12……..………..…………………………………………………………………………...15

Supplemental Table 13……..………..…………………………………………………………………………...16

Supplemental Figure 1…………………………………………………………………………………………...17

Supplemental Figure 2…………………………………………………………………………………………...18

Supplemental Figure 3………………………………………………………………………………………...…19

Supplemental Figure 4………………………………………………………………………………………...…20

Supplemental Figure 5………………………………………………………………………………………...…21

Supplemental Figure 6………………………………………………………………………………………...…22

Supplemental Figure 7………………………………………………………………………………………...…23

Supplemental Figure 8………………………………………………………………………………………...…24

Supplemental Figure 9………………………………………………………………………………………...…25

Supplemental Table 1. ICD 9 & ICD 10 Codes Used to Identify Pregnant Sample and Define Maternal Mental Health Outcomes

| **Pregnancy Identifiers** | | | | |
| --- | --- | --- | --- | --- |
| **Outcome** | | **ICD-10** | **ICD-9** | **Description** |
| Abortion | | O03- O03.9 | 634-634.92 | Spontaneous abortion |
|  | | 635.9-635.92, 636.9-636.92, 637.9-637.92, | Z33.2 | Encounter for elective termination of pregnancy |
|  | | 635-635.82, 636-636.82, 637-637.82 | O04- O04.89 | Complications following (induced) termination of pregnancy |
|  | | 638-638.9 | O07-O07.4 | Failed attempted termination of pregnancy |
| Labor and Delivery | | 641-641.93, 644.1-644.13, 645-645.23, 646-646.03, 651-651.93, 652-652.93, 653-653.93, 654-654.94, 655-655.93, 656-656.93, 657-657.03, 658-658.93, 678-678.13, 679.00, 679.03, 679.1-679.14  V91-V91.99 | O30- O36.9X9, O40-  O48.1 | Maternal care related to the fetus and amniotic cavity and possible delivery problems |
|  | | 644-644.03, 644.2-644.21,  649.8-649.82, 659-659.33, 659.7-659.93, 660-660.93, 661-661.93, 662-662.33, 663-663.93, 664-664.94, 665-665.94, 666-666.34, 667-667.14, 668-668.94, 669-669.14, 669.4-669.61, 669.80-669.94, 679.01-679.02 | O60- O77.9 | Complications of labor and delivery |
|  | | 650,  669.7-669.71 | O80-82 | Encounter for delivery |
|  | | 669.3-669.34, 670-670.84, 671.4-671.44, 671.52,  672-672.04,  673-673.84,  674.1-674.94, 675-675.94,  676-676.94, 679.04  *Many nested elsewhere in preg comp* | O85- O92.79 | Complications predominantly related to the puerperium |
|  | | V23-V23.9,  659.4-659.63 | O09- O09.93 | Supervision of high-risk pregnancy |
|  | | 642-642.94, | O10- O16.9 | Edema, proteinuria, and hypertensive disorders in pregnancy, childbirth and the puerperium |
|  | | 640-640.93, 643-643.93, 646.1-646.93,  648-648.94,  649.5-649.73, 669.2-669.24,  671-671.33,  671.5-671.51, 671.53-671.94  796.5 | O20- O29.93 | Other maternal disorders predominantly related to pregnancy |
|  | | 677 | O94 | Sequelae of complication of pregnancy, childbirth, and the puerperium |
|  | | 647-647.94 | O98- O98.93 | Maternal infectious and parasitic diseases classifiable elsewhere but complicating pregnancy, childbirth and the puerperium |
|  | | 674-674.04, 648-648.94, 649-649.44 | O99- O99.893 | Other maternal diseases classifiable elsewhere but complicating pregnancy, childbirth and the puerperium |
|  | | 648-648.94 | O9A- O9A.53 | Maternal malignant neoplasms, traumatic injuries and abuse classifiable elsewhere but complicating pregnancy, childbirth and the puerperium |
| Live Births | | V27.0 | Z37.0 | Single live birth |
|  | | V27.2 | Z37.2 | Twins, both liveborn |
|  | | V27.5 | Z37.5- Z37.59 | Other multiple births, all liveborn |
|  | | V30-V39.2 | Z38- Z38.8 | Liveborn infants according to place of birth and type of delivery |
| **Mental Health Conditions** | | | | |
| **Outcome** | **Condition** | **ICD 10** | **ICD-9** | **Description** |
| PMAD | Depression | F32 | 296.2, 311 | Depressive episode |
|  |  | F33 | 296.3 | Major depressive disorder, recurrent |
|  |  | F34.1 | 300.4 | Dysthymic disorder |
|  |  | F41.8 |  | Other specified anxiety disorders |
|  |  | F43.20 |  | Adjustment disorder, unspecified |
|  |  | F43.21 |  | Adjustment disorder with depressed mood |
|  |  | F43.23 |  | Adjustment disorder with mixed anxiety and depressed mood |
|  |  | F43.25 |  | Adjustment disorder with mixed disturbance of emotions and conduct |
|  |  | F43.29 |  | Adjustment disorder with other symptoms |
|  |  | F45.89 |  | N/A |
|  |  | F45.89 |  | N/A |
|  |  | F53.0 |  | Postpartum depression |
|  |  | F06.31 |  | Mood disorder due to known physiological condition with depressive features |
|  |  | F06.32 |  | Mood disorder due to known physiological condition with major depressive-like episode |
|  | Anxiety | F06.4 | 293.84 | Anxiety disorder due to known physiological condition |
|  |  | F40 - F48.9 | 300.00-300.9, 308, 309.81 | Anxiety, dissociative, stress-related, somatoform and other nonpsychotic mental disorders |
|  |  | F68.1 |  | Factitious disorder imposed on self |
|  |  | F93.8 | 313.0-313.22. 313.3, 313.82-313.89 | Other childhood emotional disorders |
|  |  | F95.0 |  | Transient tic disorder |
|  |  | F99.0 |  | N/A |
|  |  | R45.7 | 308.9 | State of emotional shock and stress, unspecified |
|  |  | R46.89 |  | Other symptoms and signs involving appearance and behavior |
| SMI | Bipolar | F30.10 | 296 | Manic episode without psychotic symptoms, unspecified |
|  |  | F30.11 | 296.01 | Manic episode without psychotic symptoms, mild |
|  |  | F30.12 | 296.02 | Manic episode without psychotic symptoms, moderate |
|  |  | F30.13 | 296.03 | Manic episode, severe, without psychotic symptoms |
|  |  | F30.2 | 296.04 | Manic episode, severe with psychotic symptoms |
|  |  | F30.3 | 296.05 | Manic episode in partial remission |
|  |  | F30.4 | 296.06 | Manic episode in full remission |
|  |  | F30.8 |  | Other manic episodes |
|  |  | F30.9 |  | Manic episode, unspecified |
|  |  | F31 | 296.40-296.7 | Bipolar disorder |
|  |  | F32.89 |  | Other specified depressive episodes |
|  |  | F34.81 |  | Disruptive mood dysregulation disorder |
|  |  | F39 |  | Unspecified mood [affective] disorder |
|  | Psychotic Disorders | F06.0 | 293.81-293.82 | Psychotic disorder with hallucinations due to known physiological condition |
|  |  | F06.2 |  | Psychotic disorder with delusions due to known physiological condition |
|  |  | F20.89 | 295.00-295.05 | Other schizophrenia |
|  |  | F20.0 | 295.30-295.35 | Paranoid schizophrenia |
|  |  | F20.1 | 295.10-295.15 | Disorganized schizophrenia |
|  |  | F20.2 | 295.20-295.25 | Catatonic schizophrenia |
|  |  | F20.5 | 295.60-295.65 | Residual schizophrenia |
|  |  | F20.81 | 295.40-295.45 | Schizophreniform disorder |
|  |  | F20.89 | 295.50-295.55, 295.80-295.85 | Other schizophrenia |
|  |  | F20.9 | 295.90-295.95 | Schizophrenia, unspecified |
|  |  | F22 | 297.0-297.8 | Delusional disorders |
|  |  | F23 | 297.9 | Brief psychotic disorder |
|  |  | F24 |  | Shared psychotic disorder |
|  |  | F25.0 |  | Schizoaffective disorder, bipolar type |
|  |  | F25.8 |  | Other schizoaffective disorders |
|  |  | F25.9 | 295.70-295.75 | Schizoaffective disorder, unspecified |
|  |  | F28 |  | Other psychotic disorder not due to a substance or known physiological condition |
|  |  | F29 |  | Unspecified psychosis not due to a substance or known physiological condition |
|  |  |  | 298.0-298.9 | Other nonorganic psychoses |
|  |  | F53.1 |  | Puerperal psychosis |
| MDP | Other MH Disorders Trimester 1 | O99.341 |  | Other mental disorders complicating pregnancy, first trimester |
|  | Other MH Disorders Trimester 2 | O99.342 |  | Other mental disorders complicating pregnancy, second trimester |
|  | Other MH Disorders Trimester 3 | O99.343 |  | Other mental disorders complicating pregnancy, third trimester |
|  | Other MH Disorders at Birth | O99.344 |  | Other mental disorders complicating childbirth |
|  | Other MH Disorders During Pregnancy | O99.34 |  | Other mental disorders complicating pregnancy, childbirth, and the puerperium |
|  | Other MH Disorders Postpartum | O99.345 |  | Other mental disorders complicating the puerperium |
| Suicidal thoughts (SUI-T) | Thoughts of suicide | R45.851 | V62.84 | Suicidal Ideations |
| Substance Misuse (SUB) |  | F10 | 303.93, 305.00-305.03, | Alcohol related disorders |
|  |  |  | 357.5 | Alcoholic polyneuropathy |
|  |  |  | 425.5 | Alcoholic cardiomyopathy |
|  |  |  | 535.30, 535.31 | Alcoholic gastritis, without mention of hemorrhage |
|  |  |  | 571.0-571.3 | Alcoholic fatty liver |
|  |  |  | E860 | Accidental poisoning by alcohol not elsewhere classified |
|  |  | F11 |  | Opioid related disorders |
|  |  | F12 |  | Cannabis related disorders |
|  |  | F13 |  | Sedative, hypnotic, or anxiolytic related disorders |
|  |  | F14 |  | Cocaine related disorders |
|  |  | F15 | 304.40-304.43, 305.70-305.73 | Other stimulant related disorders |
|  |  | F16 |  | Hallucinogen related disorders |
|  |  | F17 |  | Nicotine dependence |
|  |  | F18 |  | Inhalant related disorders |
|  |  | F19 |  | Other psychoactive substance related disorders |
|  |  | F55 |  | Abuse of non-psychoactive substance |

Supplemental Table 2. Methodology used to calculate the Index of Concentration at the Extremes (ICE) for racial and economic residential inequality at the zip-code tabulation area (ZCTA) level using census data from the 2018 American Community Survey.

| **ICE Metric** | **Equation** | **Variables** |
| --- | --- | --- |
| Income | ICEq=(Taq-Tpq)/Tq, | q: ZCTA location  Taq: Number of households with an annual income $\leq$ $25,000 in location q  Tpq: Number of households with an annual income  $\geq$ $100,000 in location q  Tq: Total number of households with income data in location q |
| Race | ICEq=(Taq-(Tp1q+Tp2q))/Tq | q: ZCTA  Taq: Total number of non-Hispanic white persons in location q  Tp1q: Total number of Black persons in location q  Tp2q: Total number of non-white Hispanic persons in location q  Tq: Total number of persons with race/ethnicity data in location q |

Supplemental Table 3. Case counts for maternal mental health conditions by subgroup for acute heatwave period (lag0 to lag3)

| **Category** | **Strata** | **Heatwave Period (Lag0 to Lag3)**  **n = 141,060 days** | | | | | **Non-Heatwave Matched Periods**  **n = 423,180 days** | | | | |
| --- | --- | --- | --- | --- | --- | --- | --- | --- | --- | --- | --- |
|  |  | **PMAD** | **MDP** | **SMI** | **SUI-T** | **SUB** | **PMAD** | **MDP** | **SMI** | **SUI-T** | **SUB** |
|  |  | n (%) | n (%) | n (%) | n (%) | n (%) | n (%) | n (%) | n (%) | n (%) | n (%) |
| All ED Visits | | 392 (100.0) | 568 (100.0) | 64 (100.0) | 73 (100.0) | 365 (100.0) | 1076 (100.0) | 1462 (100.0) | 215 (100.0) | 192 (100.0) | 1174 (100.0) |
| Age (Years) | Age < 35 | 358 (91.3) | 529 (93.1) | 60 (93.7) | 69 (94.5) | 320 (87.7) | 950 (88.3) | 1321 (90.4) | 199 (92.6) | 171 (89.1) | 10 (0.9) |
|  | Age >= 35 | 34 (8.7) | 39 (6.9) | 4 (6.2) | 4 (5.5) | 45 (12.3) | 126 (11.7) | 141 (9.6) | 16 (7.4) | 21 (10.9) | 99 (8.4) |
| Race | Black | 136 (34.7) | 195 (34.3) | 35 (40.6) | 26 (35.6) | 109 (29.9) | 357 (33.2) | 527 (36.0) | 111 (51.6) | 80 (41.7) | 339 (28.9) |
|  | White | 202 (51.5) | 311 (54.8) | 25 (39.0) | 39 (53.4) | 229 (62.7) | 624 (58.0) | 808 (55.3) | 86 (40.0) | 88 (45.8) | 762 (64.9) |
|  | Other Race | 46 (11.7) | 52 (9.2) | 4 (6.2) | 8 (11.0) | 26 (7.1) | 79 (7.3) | 116 (7.9) | 17 (7.9) | 20 (10.4) | 63 (5.4) |
| Ethnicity | Hispanic | 24 (6.1) | 32 (5.6) | 0 (0.0) | 5 (6.8) | 19 (5.2) | 71 (6.6) | 80 (5.5) | 13 (6.0) | 14 (7.3) | 20 (1.7) |
|  | Non-Hispanic | 348 (88.8) | 509 (89.6) | 64 (100.0) | 68 (93.2) | 338 (92.6) | 980 (91.1) | 1331 (91.0) | 194 (90.2) | 173 (90.1) | 1127 (96.0) |
| Insurance | Medicaid | 204 (52.0) | 321 (56.5) | 42 (65.6) | 50 (68.5) | 201 (55.1) | 609 (56.6) | 894 (61.1) | 110 (51.2) | 113 (58.9) | 726 (61.8) |
|  | Private Ins. | 102 (26.0) | 116 (20.4) | 2 (3.1) | 8 (11.0) | 47 (12.9) | 285 (26.5) | 297 (20.3) | 42 (19.5) | 55 (28.6) | 138 (11.8) |
|  | Self-Pay | 56 (14.3) | 83 (14.6) | 13 (20.3) | 6 (8.2) | 88 (24.1) | 106 (9.9) | 153 (10.5) | 22 (10.2) | 9 (4.7) | 255 (21.7) |
|  | Other Ins. | 27 (6.9) | 45 (7.9) | 7 (10.9) | 9 (12.3) | 29 (7.9) | 73 (6.8) | 111 (7.6) | 37 (17.2) | 15 (7.8) | 52 (4.4) |
| RUCA | Rural | 27 (6.9) | 25 (4.4) | 0 (0.0) | 2 (2.7) | 16 (4.4) | 86 (8.0) | 102 (7.0) | 16 (7.4) | 13 (6.8) | 97 (8.3) |
|  | Suburban | 81 (20.7) | 98 (17.3) | 15 (23.4) | 12 (16.4) | 69 (18.9) | 169 (15.7) | 223 (15.3) | 48 (22.3) | 30 (15.6) | 240 (20.4) |
|  | Urban | 284 (72.4) | 445 (78.3) | 49 (76.5) | 59 (80.8) | 280 (76.7) | 821 (76.3) | 1137 (77.8) | 151 (70.2) | 149 (77.6) | 837 (71.3) |
| Geographic Region | Piedmont | 201 (51.3) | 293 (51.6) | 48 (75.0) | 37 (50.7) | 199 (54.5) | 555 (51.6) | 752 (51.4) | 123 (57.2) | 92 (47.9) | 588 (50.1) |
|  | Western | 31 (7.9) | 86 (15.1) | 4 (6.2) | 12 (16.4) | 65 (17.8) | 146 (13.6) | 172 (11.8) | 24 (11.2) | 18 (9.4) | 209 (17.8) |
|  | Coastal | 160 (40.8) | 189 (33.3) | 12 (18.7) | 24 (32.9) | 101 (27.7) | 375 (34.9) | 538 (36.8) | 68 (31.6) | 82 (42.7) | 377 (32.1) |
| ICE Race | Q1: Mostly Non-White | 106 (27.0) | 150 (26.4) | 24 (37.5) | 19 (26.0) | 127 (34.8) | 326 (30.3) | 486 (33.2) | 94 (43.7) | 77 (40.1) | 328 (27.9) |
|  | Q2 | 128 (32.7) | 168 (29.6) | 18 (28.1) | 17 (23.3) | 83 (22.7) | 262 (24.3) | 372 (25.4) | 55 (25.6) | 56 (29.2) | 301 (25.6) |
|  | Q3: Mixed -Race | 85 (21.7) | 118 (20.8) | 9 (14.1) | 14 (19.2) | 82 (22.5) | 259 (24.1) | 315 (21.5) | 38 (17.7) | 35 (18.2) | 262 (22.3) |
|  | Q4 | 55 (14.0) | 105 (18.5) | 9 (14.1) | 19 (26.0) | 50 (13.7) | 176 (16.4) | 230 (15.7) | 23 (10.7) | 22 (11.5) | 181 (15.4) |
|  | Q5: Mostly White | 18 (4.6) | 27 (4.8) | 4 (6.3) | 4 (5.5) | 23 (6.3) | 53 (4.9) | 59 (4.0) | 5 (2.3) | 2 (1.0) | 102 (8.7) |
| ICE Income | Q1: Mostly Low-Income | 69 (17.6) | 91 (16.0) | 14 (21.8) | 8 (11.0) | 68 (18.6) | 143 (13.3) | 232 (15.9) | 35 (16.3) | 26 (13.5) | 185 (15.8) |
|  | Q2 | 63 (16.1) | 74 (13.0) | 12 (18.7) | 7 (9.6) | 74 (20.3) | 180 (16.7) | 235 (16.1) | 49 (22.8) | 48 (25.0) | 248 (21.1) |
|  | Q3: Mixed-Income | 78 (19.9) | 149 (26.2) | 21 (32.8) | 20 (27.4) | 87 (23.8) | 249 (23.1) | 342 (23.4) | 60 (27.9) | 43 (22.4) | 278 (23.7) |
|  | Q4 | 117 (29.8) | 140 (24.6) | 9 (14.0) | 18 (24.7) | 88 (24.1) | 328 (30.5) | 379 (25.9) | 46 (21.4) | 52 (27.1) | 289 (24.6) |
|  | Q5: Mostly Wealthy | 65 (16.6) | 114 (20.1) | 8 (12.5) | 20 (27.4) | 48 (13.2) | 176 (16.4) | 274 (18.7) | 25 (11.6) | 23 (12.0) | 174 (14.8) |
| *PMAD=Perinatal mood and anxiety disorders; MDP=Maternal mental disorders of pregnancy; SMI=Severe mental illness; SUI-T=Suicidal thoughts; SUB=Substance misuse; ICE=Index of Concentration of Concentration at the Extremes; RUCA=Rural-Urban Commuting Area* | | | | | | | | | | | |

Supplemental Table 4. Case counts for maternal mental health conditions by subgroup for prolonged heatwave period (lag0 to lag7)

| **Category** | **Strata** | **Heatwave Period (Lag0 to Lag7)**  n = 282,120 days | | | | | **Non-Heatwave Matched Periods**  n = 846,360 days | | | | |
| --- | --- | --- | --- | --- | --- | --- | --- | --- | --- | --- | --- |
|  |  | **PMAD** | **MDP** | **SMI** | **SUI-T** | **SUB** | **PMAD** | **MDP** | **SMI** | **SUI-T** | **SUB** |
|  | | n (%) | n (%) | n (%) | n (%) | n (%) | n (%) | n (%) | n (%) | n (%) | n (%) |
| All ED Visits | | 773 (100.0) | 1106 (100.0) | 137 (100.0) | 119 (100.0) | 733 (100.0) | 2130 (100.0) | 2852 (100.0) | 407 (100.0) | 360 (100.0) | 2261 (100.0) |
| Age (Years) | Age < 35 | 688 (89.0) | 1012 (91.5) | 125 (91.2) | 111 (93.3) | 655 (89.4) | 1898 (89.1) | 2566 (90.0) | 366 (89.9) | 316 (87.8) | 2061 (91.2) |
|  | Age >= 35 | 85 (11.0) | 94 (8.5) | 12 (8.8) | 8 (6.7) | 78 (10.6) | 232 (10.9) | 286 (10.0) | 41 (10.1) | 44 (12.2) | 200 (8.8) |
| Race | Black | 292 (37.8) | 383 (34.6) | 63 (46.0) | 39 (32.8) | 235 (32.1) | 718 (33.7) | 1010 (35.4) | 192 (47.2) | 150 (41.7) | 620 (27.4) |
|  | White | 389 (50.3) | 605 (54.7) | 65 (47.4) | 60 (50.4) | 451 (61.5) | 1200 (56.3) | 1599 (56.1) | 175 (43.0) | 164 (45.6) | 1504 (66.5) |
|  | Other Race | 78 (10.1) | 96 (8.7) | 4 (2.9) | 20 (16.8) | 46 (6.3) | 177 (8.3) | 212 (7.4) | 38 (9.3) | 40 (11.1) | 119 (5.3) |
| Ethnicity | Hispanic | 42 (5.4) | 63 (5.7) | 0 (0.0) | 16 (13.4) | 23 (3.1) | 147 (6.9) | 148 (5.2) | 27 (6.6) | 26 (7.2) | 43 (1.9) |
|  | Non-Hispanic | 698 (90.3) | 993 (89.8) | 131 (95.6) | 103 (86.6) | 695 (94.8) | 1939 (91.0) | 2624 (92.0) | 367 (90.2) | 323 (89.7) | 2179 (96.4) |
| Insurance | Medicaid | 407 (52.7) | 616 (55.7) | 91 (66.4) | 76 (63.9) | 420 (57.3) | 1195 (56.1) | 1759 (61.7) | 208 (51.1) | 221 (61.4) | 1382 (61.1) |
|  | Private Ins. | 217 (28.1) | 243 (22.0) | 14 (10.2) | 15 (12.6) | 96 (13.1) | 552 (25.9) | 553 (19.4) | 72 (17.7) | 84 (23.3) | 269 (11.9) |
|  | Self-Pay | 95 (12.3) | 161 (14.6) | 18 (13.1) | 19 (16.0) | 175 (23.9) | 231 (10.8) | 301 (10.6) | 57 (14.0) | 21 (5.8) | 505 (22.3) |
|  | Other Ins. | 51 (6.6) | 83 (7.5) | 14 (10.2) | 9 (7.6) | 42 (5.7) | 144 (6.8) | 228 (8.0) | 65 (16.0) | 34 (9.4) | 102 (4.5) |
| RUCA | Rural | 44 (5.7) | 52 (4.7) | 0 (0.0) | 4 (3.4) | 36 (4.9) | 191 (9.0) | 194 (6.8) | 35 (8.6) | 22 (6.1) | 226 (10.0) |
|  | Suburban | 157 (20.3) | 166 (15.0) | 30 (21.8) | 15 (12.6) | 135 (18.4) | 356 (16.7) | 431 (15.1) | 99 (24.3) | 65 (18.1) | 435 (19.2) |
|  | Urban | 572 (73.9) | 888 (80.2) | 107 (78.1) | 100 (84.0) | 562 (76.6) | 1583 (74.3) | 2227 (78.1) | 273 (67.1) | 273 (75.8) | 1600 (70.8) |
| Geographic Region | Piedmont | 399 (51.6) | 596 (53.9) | 90 (65.7) | 68 (57.1) | 389 (53.1) | 1086 (51.0) | 1447 (50.7) | 216 (53.1) | 171 (47.5) | 1141 (50.5) |
|  | Western | 72 (9.3) | 157 (14.2) | 13 (9.5) | 13 (10.9) | 135 (18.4) | 299 (14.0) | 365 (12.8) | 47 (11.5) | 38 (10.6) | 408 (18.0) |
|  | Coastal | 302 (39.1) | 353 (31.9) | 34 (24.8) | 38 (31.9) | 209 (28.5) | 745 (35.0) | 1040 (36.5) | 144 (35.4) | 151 (41.9) | 712 (31.5) |
| ICE Race | Q1: Mostly Non-White | 251 (32.4) | 339 (30.6) | 52 (37.9) | 33 (27.7) | 246 (33.5) | 681 (32.0) | 919 (32.2) | 178 (43.7) | 135 (37.5) | 612 (27.1) |
|  | Q2 | 231 (29.8) | 305 (27.5) | 40 (29.1) | 28 (23.5) | 159 (21.6) | 514 (24.1) | 743 (26.1) | 116 (28.5) | 105 (29.2) | 576 (25.5) |
|  | Q3: Mixed-Race | 160 (20.6) | 230 (20.7) | 23 (16.7) | 26 (21.8) | 151 (20.6) | 480 (22.5) | 612 (21.5) | 57 (14.0) | 63 (17.5) | 488 (21.6) |
|  | Q4 | 105 (13.5) | 175 (15.8) | 16 (11.6) | 28 (23.5) | 130 (17.7) | 337 (15.8) | 446 (15.6) | 46 (11.3) | 49 (13.6) | 371 (16.4) |
|  | Q5: Mostly White | 26 (3.3) | 57 (5.1) | 6 (4.3) | 4 (3.3) | 47 (6.4) | 118 (5.5) | 131 (4.6) | 10 (2.5) | 8 (2.2) | 214 (9.5) |
| ICE Income | Q1: Mostly Low-Income | 141 (18.2) | 171 (15.4) | 26 (18.9) | 11 (9.2) | 144 (19.6) | 306 (14.4) | 432 (15.1) | 76 (18.7) | 51 (14.2) | 365 (16.1) |
|  | Q2 | 122 (15.7) | 141 (12.7) | 22 (16.0) | 11 (9.2) | 126 (17.1) | 370 (17.4) | 444 (15.6) | 83 (20.4) | 77 (21.4) | 490 (21.7) |
|  | Q3: Mixed-Income | 145 (18.7) | 286 (25.8) | 44 (32.1) | 28 (23.5) | 203 (27.6) | 475 (22.3) | 682 (23.9) | 98 (24.1) | 80 (22.2) | 545 (24.1) |
|  | Q4 | 231 (29.8) | 305 (27.5) | 29 (21.1) | 33 (27.7) | 167(22.7) | 637 (29.9) | 782 (27.4) | 102 (25.1) | 105 (29.2) | 543 (24.0) |
|  | Q5: Mostly Wealthy | 134 (17.3) | 203 (18.3) | 16 (11.6) | 36 (30.2) | 93 (12.6) | 342 (16.1) | 511 (17.9) | 48 (11.8) | 47 (13.1) | 318 (14.1) |
| *PMAD=Perinatal mood and anxiety disorders; MDP=Maternal mental disorders of pregnancy; SMI=Severe mental illness; SUI-T=Suicidal thoughts; SUB=Substance misuse; ICE=Index of Concentration of Concentration at the Extremes; RUCA=Rural-Urban Commuting Area* | | | | | | | | | | | |

Supplemental Table 5. Daily relative risk (RR) values, 95% confidence intervals (CIs), and *p*-value estimates for heatwave days (lag0) and the seven consecutive days following (lag1 to lag7) for maternal mental health conditions.

| **Lag Day** | **PMAD RR (95%CI)** | **p** | **MDP RR (95%CI)** | **p** | **SMI RR (95%CI)** | **p** | **SUIT RR (95%CI)** | **p** | **SUB RR (95%CI)** | **p** |
| --- | --- | --- | --- | --- | --- | --- | --- | --- | --- | --- |
| lag0 | 0.93 (0.81-1.07) | 0.29 | 1.04 (0.92-1.17) | 0.55 | 0.98 (0.81-1.18) | 0.82 | 0.94 (0.77-1.16) | 0.57 | 0.98 (0.85-1.11) | 0.71 |
| lag1 | 1.11 (0.95-1.29) | 0.19 | 1.03 (0.90-1.18) | 0.65 | 1.08 (0.87-1.33) | 0.50 | 1.22 (0.97-1.54) | 0.080 | 0.94 (0.81-1.09) | 0.41 |
| lag2 | 0.91 (0.79-1.06) | 0.21 | 0.90 (0.79-1.02) | 0.10 | 0.90 (0.73-1.10) | 0.30 | 0.94 (0.75-1.17) | 0.56 | 1.10 (0.96-1.26) | 0.18 |
| lag3 | 0.88 (0.76-1.02) | 0.081 | 0.99 (0.87-1.12) | 0.87 | 0.83 (0.68-1.01) | 0.059 | 0.78 (0.63-0.98) | 0.030 | 0.96 (0.84-1.10) | 0.57 |
| lag4 | 1.02 (0.88-1.17) | 0.81 | 0.98 (0.87-1.12) | 0.81 | 1.46 (1.21-1.76) | 0.0001 | 1.10 (0.88-1.36) | 0.41 | 1.12 (0.98-1.29) | 0.090 |
| lag5 | 1.00 (0.87-1.15) | 0.97 | 1.04 (0.91-1.17) | 0.58 | 0.84 (0.69-1.02) | 0.085 | 0.97 (0.78-1.20) | 0.76 | 0.83 (0.73-0.96) | 0.010 |
| lag6 | 1.14 (0.99-1.31) | 0.072 | 0.98 (0.86-1.11) | 0.70 | 1.34 (1.11-1.63) | 0.0028 | 0.85 (0.68-1.06) | 0.15 | 1.12 (0.97-1.29) | 0.11 |
| lag7 | 0.92 (0.82-1.05) | 0.21 | 0.96 (0.86-1.07) | 0.44 | 0.80 (0.67-0.96) | 0.015 | 1.28 (1.07-1.54) | 0.0069 | 1.05 (0.93-1.18) | 0.43 |
| *PMAD=Perinatal mood and anxiety disorders; MDP=Maternal mental disorders of pregnancy; SMI=Severe mental illness; SUI-T=Suicidal thoughts; SUB=Substance misuse* | | | | | | | | | | |

Supplemental Table 6. Cumulative relative risk (RR) estimates, 95% confidence intervals (CIs), and corresponding *p*-values for acute heatwave periods (lag0 to lag3) for maternal mental health outcomes.

|  | | **PMAD** | | **MDP** | | **SMI** | | **SUIT** | | **SUB** | |
| --- | --- | --- | --- | --- | --- | --- | --- | --- | --- | --- | --- |
| **Category** | **Subgroup** | **RR (95% CI^7^)** | **p^a^** | **RR (95% CI)** | **p^a^** | **RR (95% CI)** | **p^a^** | **RR (95% CI)** | **p^a^** | **RR (95% CI)** | **p^a^** |
| Age | >= 35 | 0.96 (0.91-1.01) | 0.13 | 1.00 (0.95-1.06) | 0.94 | 1.81 (1.45-2.25) | < 0.0001 | 1.14 (0.98-1.33) | 0.08 | 1.09 (1.02-1.18) | 0.01 |
|  | < 35 | 0.95 (0.91-0.98) | 0.0018 | 1.00 (0.97-1.03) | 0.96 | 1.12 (1.07-1.17) | < 0.0001 | 1.07 (1.01-1.13) | 0.023 | 1.01 (0.97-1.04) | 0.68 |
| Race | Black | 0.88 (0.85-0.92) | < 0.0001 | 1.00 (0.97-1.04) | 0.82 | 1.14 (1.08-1.20) | < 0.0001 | 1.08 (1.03-1.15) | 0.0043 | 0.98 (0.93-1.02) | 0.30 |
|  | White | 1.00 (0.95-1.04) | 0.88 | 0.99 (0.95-1.03) | 0.55 | 1.21 (1.13-1.30) | < 0.0001 | 1.02 (0.95-1.10) | 0.50 | 1.03 (0.99-1.07) | 0.11 |
|  | Other Race | 1.05 (1.01-1.09) | 0.0062 | 1.03 (0.99-1.08) | 0.11 | 1.05 (0.96-1.14) | 0.28 | 1.02 (0.97-1.07) | 0.43 | 1.10 (1.04-1.16) | 0.0007 |
| Insurance | Medicaid | 0.90 (0.87-0.93) | < 0.0001 | 0.96 (0.93-1.00) | 0.028 | 1.21 (1.13-1.29) | < 0.0001 | 1.17 (1.06-1.29) | 0.0014 | 1.04 (1.00-1.08) | 0.030 |
|  | Private | 1.02 (0.99-1.05) | 0.18 | 1.03 (1.00-1.06) | 0.055 | 1.24 (1.14-1.35) | < 0.0001 | 1.19 (1.12-1.25) | < 0.0001 | 0.96 (0.93-1.00) | 0.050 |
|  | Self Pay | 1.01 (0.96-1.06) | 0.65 | 1.02 (0.96-1.09) | 0.51 | 1.03 (0.93-1.15) | 0.57 | 0.95 (0.88-1.02) | 0.16 | 0.96 (0.91-1.02) | 0.14 |
|  | Other | 1.52 (1.25-1.85) | < 0.0001 | 0.97 (0.92-1.04) | 0.42 | 1.02 (0.95-1.10) | 0.61 | 0.65 (0.62-0.68) | < 0.0001 | 0.99 (0.94-1.05) | 0.69 |
| RUCA | Urban | 0.96 (0.91-1.00) | 0.050 | 0.99 (0.95-1.03) | 0.53 | 1.07 (1.01-1.14) | 0.023 | 1.06 (0.98-1.14) | 0.15 | 1.00 (0.96-1.05) | 0.85 |
|  | Suburban | 1.01 (0.94-1.09) | 0.81 | 1.05 (0.98-1.13) | 0.14 | 1.35 (1.16-1.57) | < 0.0001 | 1.10 (0.96-1.24) | 0.15 | 1.01 (0.94-1.09) | 0.72 |
|  | Rural* | 0.94 (0.80-1.09) | 0.39 | 1.08 (0.97-1.21) | 0.14 | NA | NA | 0.96 (0.86-1.07) | 0.46 | 1.17 (1.02-1.33) | 0.020 |
| Region | Western | 1.08 (1.00-1.17) | 0.047 | 1.00 (0.92-1.09) | 0.97 | 1.14 (0.97-1.34) | 0.11 | 1.20 (0.87-1.66) | 0.26 | 1.11 (1.02-1.21) | 0.013 |
|  | Coastal | 0.88 (0.84-0.92) | < 0.0001 | 1.02 (0.97-1.07) | 0.36 | 1.22 (1.13-1.32) | < 0.0001 | 1.14 (1.01-1.30) | 0.037 | 1.02 (0.95-1.09) | 0.59 |
|  | Piedmont | 0.98 (0.92-1.05) | 0.61 | 0.99 (0.94-1.05) | 0.70 | 1.12 (1.03-1.21) | 0.0057 | 1.19 (1.02-1.39) | 0.028 | 1.02 (0.96-1.08) | 0.51 |
| ICE Race | Q1 | 0.90 (0.84-0.95) | 0.00030 | 1.01 (0.94-1.08) | 0.83 | 1.09 (0.98-1.20) | 0.10 | 1.14 (1.00-1.31) | 0.056 | 1.02 (0.94-1.11) | 0.59 |
|  | Q2 | 0.98 (0.89-1.07) | 0.58 | 1.04 (0.96-1.11) | 0.33 | 1.17 (1.01-1.36) | 0.032 | 0.97 (0.86-1.09) | 0.56 | 0.98 (0.90-1.06) | 0.59 |
|  | Q3 | 0.95 (0.88-1.03) | 0.20 | 0.94 (0.87-1.00) | 0.066 | 1.36 (1.10-1.67) | 0.0040 | 1.63 (1.01-2.61) | 0.043 | 1.01 (0.94-1.08) | 0.83 |
|  | Q4 | 1.04 (0.94-1.16) | 0.45 | 1.04 (0.96-1.12) | 0.37 | 1.81 (1.35-2.44) | < 0.0001 | 1.04 (0.86-1.24) | 0.70 | 1.10 (1.00-1.21) | 0.041 |
|  | Q5* | 1.10 (0.94-1.28) | 0.21 | 1.23 (0.98-1.54) | 0.069 | NA | NA | NA | NA | 1.14 (1.03-1.25) | 0.0094 |
| ICE Income | Q1 | 1.02 (0.93-1.11) | 0.67 | 1.11 (0.99-1.25) | 0.084 | 0.84 (0.77-0.92) | 0.00020 | 0.66 (0.60-0.72) | < 0.0001 | 0.95 (0.89-1.03) | 0.21 |
|  | Q2 | 0.95 (0.85-1.06) | 0.36 | 1.01 (0.93-1.10) | 0.74 | 1.42 (1.19-1.68) | < 0.0001 | 1.15 (1.04-1.26) | 0.0048 | 1.08 (0.97-1.20) | 0.14 |
|  | Q3 | 0.88 (0.82-0.95) | 0.00080 | 0.94 (0.87-1.02) | 0.12 | 1.36 (1.11-1.68) | 0.0030 | 1.39 (1.02-1.90) | 0.034 | 1.13 (1.04-1.24) | 0.0064 |
|  | Q4 | 0.98 (0.90-1.07) | 0.65 | 1.05 (0.97-1.13) | 0.19 | 1.22 (0.92-1.60) | 0.16 | 1.35 (1.05-1.73) | 0.017 | 0.96 (0.89-1.03) | 0.22 |
|  | Q5 | 1.03 (0.93-1.14) | 0.60 | 1.06 (0.97-1.17) | 0.19 | 1.71 (1.17-2.50) | 0.0052 | 1.07 (0.96-1.21) | 0.22 | 1.08 (0.94-1.23) | 0.27 |
| *PMAD=Perinatal mood and anxiety disorders; MDP=Maternal mental disorders of pregnancy; SMI=Severe mental illness; SUI-T=Suicidal thoughts; SUB=Substance misuse; RR= Relative risk; CI=Confidence interval; ICE=Index of Concentration of Concentration at the Extremes; RUCA=Rural-Urban Commuting Area*  **Results for select outcomes excluded (NA) due to insufficient sample size*  **^a^***The p-values test the null hypothesis that heatwaves have no effect on maternal mental health outcomes for each strata (e.g., Age, Race)* | | | | | | | | | | | |

Supplemental Table 7. *p*-values for effect modification (*p*-EM) across subgroups during acute heatwave periods (lag0 to lag3)

|  | | **PMAD** | | **MDP** | | **SMI** | | **SUIT** | | **SUB** | |
| --- | --- | --- | --- | --- | --- | --- | --- | --- | --- | --- | --- |
| **Category** | **Subgroup** | **RR**  **(95% CI^7^)** | **p-EM^a^** | **RR**  **(95% CI)** | **p-EM^a^** | **RR**  **(95% CI)** | **p-EM^a^** | **RR**  **(95% CI)** | **p-EM^a^** | **RR**  **(95% CI)** | **p-EM^a^** |
| Age | < 35 | 0.95 (0.91-0.98) | Ref. | 1.00 (0.97-1.03) | Ref. | 1.12 (1.07-1.17) | Ref. | 1.07 (1.01-1.13) | Ref. | 1.01 (0.97-1.04) | Ref. |
|  | >= 35 | 0.96 (0.91-1.01) | 0.69 | 1.00 (0.95-1.06) | 0.96 | 1.81 (1.45-2.25) | <0.0001 | 1.14 (0.98-1.33) | 0.39 | 1.09 (1.02-1.18) | 0.043 |
| Race | White | 1.00 (0.95-1.04) | Ref. | 0.99 (0.95-1.03) | Ref. | 1.21 (1.13-1.30) | Ref. | 1.02 (0.95-1.10) | Ref. | 1.03 (0.99-1.07) | Ref. |
|  | Black | 0.88 (0.85-0.92) | <0.0001 | 1.00 (0.97-1.04) | 0.56 | 1.14 (1.08-1.20) | 0.15 | 1.08 (1.03-1.15) | 0.22 | 0.98 (0.93-1.02) | 0.071 |
|  | Other Race | 1.05 (1.01-1.09) | 0.064 | 1.03 (0.99-1.08) | 0.12 | 1.05 (0.96-1.14) | 0.0095 | 1.02 (0.97-1.07) | 0.90 | 1.10 (1.04-1.16) | 0.05 |
| Insurance | Private | 1.02 (0.99-1.05) | Ref. | 1.03 (1.00-1.06) | Ref. | 1.24 (1.14-1.35) | Ref. | 1.19 (1.12-1.25) | Ref. | 0.96 (0.93-1.00) | Ref. |
|  | Medicaid | 0.90 (0.87-0.93) | <0.0001 | 0.96 (0.93-1.00) | 0.0036 | 1.21 (1.13-1.29) | 0.56 | 1.17 (1.06-1.29) | 0.82 | 1.04 (1.00-1.08) | 0.0044 |
|  | Other | 1.52 (1.25-1.85) | <0.0001 | 0.97 (0.92-1.04) | 0.83 | 1.02 (0.95-1.10) | <0.0001 | 0.65 (0.62-0.68) | <0.0001 | 0.99 (0.94-1.05) | 0.44 |
|  | Self Pay | 1.01 (0.96-1.06) | 0.74 | 1.02 (0.96-1.09) | 0.95 | 1.03 (0.93-1.15) | 0.00028 | 0.95 (0.88-1.02) | <0.0001 | 0.96 (0.91-1.02) | 0.88 |
| RUCA | Urban | 0.96 (0.91-1.00) | Ref. | 0.99 (0.95-1.03) | Ref. | 1.07 (1.01-1.14) | Ref. | 1.06 (0.98-1.14) | Ref. | 1.00 (0.96-1.05) | Ref. |
|  | Rural* | 0.94 (0.80-1.09) | 0.79 | 1.08 (0.97-1.21) | 0.11 | NA | NA | 0.96 (0.86-1.07) | 0.15 | 1.17 (1.02-1.33) | 0.03 |
|  | Suburban | 1.01 (0.94-1.09) | 0.22 | 1.05 (0.98-1.13) | 0.0050 | 1.35 (1.16-1.57) | 0.11 | 1.10 (0.96-1.24) | 0.63 | 1.01 (0.94-1.09) | 0.82 |
| Region | Piedmont | 0.98 (0.92-1.05) | Ref. | 0.99 (0.94-1.05) | Ref. | 1.12 (1.03-1.21) | Ref. | 1.19 (1.02-1.39) | Ref. | 1.02 (0.96-1.08) | Ref. |
|  | Western | 1.08 (1.00-1.17) | 0.064 | 1.00 (0.92-1.09) | 0.068 | 1.14 (0.97-1.34) | 0.10 | 1.20 (0.87-1.66) | 0.94 | 1.11 (1.02-1.21) | 0.10 |
|  | Coastal | 0.88 (0.84-0.92) | 0.0054 | 1.02 (0.97-1.07) | 0.10 | 1.22 (1.13-1.32) | 0.38 | 1.14 (1.01-1.30) | 0.70 | 1.02 (0.95-1.09) | 0.97 |
| ICE Race | Q5* | 1.10 (0.94-1.28) | Ref. | 1.23 (0.98-1.54) | Ref. | NA | NA | NA | NA | 1.14 (1.03-1.25) | Ref. |
|  | Q4 | 1.04 (0.94-1.16) | 0.55 | 1.04 (0.96-1.12) | 0.15 | 1.81 (1.35-2.44) | NA | 1.04 (0.86-1.24) | NA | 1.10 (1.00-1.21) | 0.64 |
|  | Q3 | 0.95 (0.88-1.03) | 0.091 | 0.94 (0.87-1.00) | 0.022 | 1.36 (1.10-1.67) | NA | 1.63 (1.01-2.61) | NA | 1.01 (0.94-1.08) | 0.043 |
|  | Q2 | 0.98 (0.89-1.07) | 0.17 | 1.04 (0.96-1.11) | 0.15 | 1.17 (1.01-1.36) | NA | 0.97 (0.86-1.09) | NA | 0.98 (0.90-1.06) | 0.020 |
|  | Q1 | 0.90 (0.84-0.95) | 0.013 | 1.01 (0.94-1.08) | 0.092 | 1.09 (0.98-1.20) | NA | 1.63 (1.01-2.61) | NA | 1.02 (0.94-1.11) | 0.10 |
| ICE Income | Q5 | 1.03 (0.93-1.14) | Ref. | 1.06 (0.97-1.17) | Ref. | 1.71 (1.17-2.50) | Ref. | 1.07 (0.96-1.21) | Ref. | 1.08 (0.94-1.23) | Ref. |
|  | Q4 | 0.98 (0.90-1.07) | 0.49 | 1.05 (0.97-1.13) | 0.83 | 1.22 (0.92-1.60) | 0.15 | 1.35 (1.05-1.73) | 0.10 | 0.96 (0.89-1.03) | 0.12 |
|  | Q3 | 0.88 (0.82-0.95) | 0.020 | 0.94 (0.87-1.02) | 0.047 | 1.36 (1.11-1.68) | 0.30 | 1.39 (1.02-1.90) | 0.12 | 1.13 (1.04-1.24) | 0.53 |
|  | Q2 | 0.95 (0.85-1.06) | 0.31 | 1.01 (0.93-1.10) | 0.45 | 1.42 (1.19-1.68) | 0.36 | 1.15 (1.04-1.26) | 0.39 | 1.08 (0.97-1.20) | 0.98 |
|  | Q1 | 1.02 (0.93-1.11) | 0.89 | 1.11 (0.99-1.25) | 0.58 | 0.84 (0.77-0.92) | 0.00035 | 0.66 (0.60-0.72) | <0.0001 | 0.95 (0.89-1.03) | 0.11 |
| *PMAD=Perinatal mood and anxiety disorders; MDP=Maternal mental disorders of pregnancy; SMI=Severe mental illness; SUI-T=Suicidal thoughts; SUB=Substance misuse; RR= Relative risk; CI=Confidence interval; RUCA=Rural-Urban Commuting Area Codes; ICE=Index of Concentration of Extremes*  **Results for select outcomes excluded (NA) due to insufficient sample size*  ^a^*p-EM=*^a^*p-EM= p-value used to assess effect modification (EM). Effect modification occurs when the relationship between the heatwaves and maternal health outcome varies across levels of a third variable (e.g., Age, Race, Region) and helps determine whether there is a significant difference in the effects between these groups. The statistic is calculated as: W = ((Beta_1 - Beta_2)^2) / (SE_Beta_1^2 + SE_Beta_2^2) Where: Beta_1 is the coefficient estimate for the first group (e.g., over age 35)), Beta_2 is the coefficient estimate for the reference group, SE_Beta_1 is the standard error of the coefficient for the first group, SE_Beta_2 is the standard error of the coefficient for the reference group.p-EM=p-value for Effect modification* | | | | | | | | | | | |

Supplemental Table 8. Cumulative relative risk (RR) estimates, 95% confidence intervals (CIs), and corresponding *p*-values for prolonged heatwave periods (lag0 to lag7) for maternal mental health outcomes.

|  | | **PMAD** | | **MDP** | | **SMI** | | **SUIT** | | **SUB** | |
| --- | --- | --- | --- | --- | --- | --- | --- | --- | --- | --- | --- |
| **Category** | **Subgroup** | **RR (95% CI^7^)** | **p^a^** | **RR (95% CI)** | **p^a^** | **RR (95% CI)** | **p^a^** | **RR (95% CI)** | **p^a^** | **RR (95% CI)** | **p^a^** |
| Age | >= 35 | 1.02 (0.98-1.05) | 0.31 | 1.01 (0.98-1.04) | 0.42 | 1.48 (1.34-1.63) | < 0.0001 | 1.14 (1.08-1.22) | < 0.0001 | 1.05 (1.00-1.09) | 0.036 |
|  | < 35 | 0.99 (0.97-1.01) | 0.17 | 0.99 (0.97-1.01) | 0.18 | 1.02 (0.98-1.05) | 0.29 | 1.00 (0.97-1.03) | 0.95 | 1.01 (0.99-1.03) | 0.21 |
| Race | Black | 0.97 (0.95-0.99) | 0.0068 | 1.00 (0.98-1.02) | 0.93 | 1.06 (1.01-1.10) | 0.0084 | 1.04 (1.00-1.09) | 0.04 | 1.00 (0.98-1.03) | 0.78 |
|  | White | 1.00 (0.97-1.02) | 0.73 | 0.99 (0.97-1.01) | 0.16 | 1.12 (1.06-1.17) | < 0.0001 | 1.02 (0.99-1.06) | 0.23 | 1.02 (1.00-1.04) | 0.092 |
|  | Other Race | 1.05 (1.01-1.09) | 0.0062 | 1.03 (0.99-1.08) | 0.11 | 1.05 (0.96-1.14) | 0.28 | 1.02 (0.97-1.07) | 0.43 | 1.10 (1.04-1.16) | 0.00070 |
| Insurance | Medicaid | 0.97 (0.94-0.99) | 0.0042 | 0.99 (0.97-1.01) | 0.45 | 1.05 (1.02-1.09) | 0.0010 | 1.02 (0.98-1.05) | 0.32 | 1.03 (1.01-1.06) | 0.0036 |
|  | Private | 1.02 (0.99-1.05) | 0.18 | 1.03 (1.00-1.06) | 0.055 | 1.24 (1.14-1.35) | < 0.0001 | 1.19 (1.12-1.25) | < 0.0001 | 0.96 (0.93-1.00) | 0.050 |
|  | Self Pay | 1.05 (1.01-1.09) | 0.0083 | 0.94 (0.90-0.98) | 0.0016 | 0.99 (0.93-1.05) | 0.70 | 0.92 (0.85-1.00) | 0.048 | 1.01 (0.98-1.04) | 0.45 |
|  | Other | 1.11 (1.07-1.16) | < 0.0001 | 0.99 (0.96-1.03) | 0.73 | 0.90 (0.87-0.93) | < 0.0001 | 1.03 (0.95-1.12) | 0.41 | 1.10 (1.02-1.19) | 0.010 |
| RUCA | Urban | 0.99 (0.96-1.01) | 0.27 | 0.98 (0.96-1.00) | 0.055 | 1.02 (0.98-1.06) | 0.35 | 1.01 (0.96-1.06) | 0.67 | 0.99 (0.97-1.02) | 0.66 |
|  | Suburban | 1.03 (0.98-1.07) | 0.21 | 1.03 (0.99-1.07) | 0.11 | 1.05 (0.98-1.13) | 0.15 | 1.08 (1.01-1.16) | 0.024 | 1.06 (1.01-1.11) | 0.012 |
|  | Rural* | 1.08 (0.94-1.23) | 0.26 | 1.00 (0.94-1.06) | 0.99 | NA | NA | 1.51 (1.17-1.96) | 0.0015 | 1.08 (1.01-1.14) | 0.015 |
| Region | Western | 1.09 (1.02-1.17) | 0.0086 | 0.98 (0.93-1.03) | 0.46 | 0.95 (0.86-1.06) | 0.37 | 1.09 (0.91-1.31) | 0.33 | 1.05 (1.00-1.11) | 0.049 |
|  | Coastal | 0.99 (0.96-1.01) | 0.33 | 1.01 (0.98-1.03) | 0.52 | 1.06 (0.99-1.14) | 0.10 | 1.02 (0.98-1.06) | 0.44 | 1.00 (0.97-1.04) | 0.87 |
|  | Piedmont | 0.98 (0.95-1.01) | 0.23 | 0.98 (0.95-1.00) | 0.094 | 1.06 (1.01-1.12) | 0.013 | 1.08 (1.00-1.18) | 0.049 | 1.02 (0.99-1.05) | 0.13 |
| ICE Race | Q1 | 0.98 (0.94-1.02) | 0.29 | 0.99 (0.96-1.03) | 0.72 | 1.00 (0.95-1.06) | 0.85 | 1.09 (1.00-1.19) | 0.047 | 1.01 (0.98-1.05) | 0.54 |
|  | Q2 | 1.02 (0.98-1.07) | 0.35 | 1.01 (0.97-1.05) | 0.58 | 1.09 (0.98-1.20) | 0.10 | 1.04 (0.94-1.14) | 0.46 | 1.00 (0.96-1.04) | 0.97 |
|  | Q3 | 0.98 (0.93-1.03) | 0.43 | 0.96 (0.92-1.00) | 0.060 | 1.14 (0.98-1.32) | 0.087 | 1.08 (0.99-1.19) | 0.087 | 1.02 (0.98-1.07) | 0.38 |
|  | Q4 | 1.05 (0.98-1.12) | 0.15 | 1.02 (0.98-1.07) | 0.36 | 1.35 (1.14-1.60) | 0.00060 | 1.00 (0.87-1.15) | 0.99 | 1.10 (1.01-1.18) | 0.021 |
|  | Q5 | 1.08 (0.98-1.19) | 0.11 | 1.04 (0.97-1.11) | 0.28 | 1.13 (0.94-1.36) | 0.18 | 1.79 (1.24-2.58) | 0.0018 | 1.06 (0.98-1.14) | 0.12 |
| ICE Income | Q1 | 1.00 (0.95-1.05) | 0.91 | 0.97 (0.93-1.01) | 0.092 | 0.9 (0.84-0.97) | 0.0048 | 0.95 (0.84-1.07) | 0.37 | 1.02 (0.97-1.07) | 0.43 |
|  | Q2 | 1.00 (0.96-1.06) | 0.86 | 1.01 (0.96-1.05) | 0.79 | 1.16 (1.06-1.27) | 0.0016 | 1.18 (1.07-1.30) | 0.00060 | 1.05 (1.00-1.11) | 0.036 |
|  | Q3 | 0.97 (0.93-1.02) | 0.21 | 0.97 (0.93-1.01) | 0.10 | 1.05 (0.95-1.14) | 0.33 | 1.19 (1.02-1.38) | 0.027 | 1.03 (0.98-1.07) | 0.25 |
|  | Q4 | 1.03 (0.98-1.08) | 0.24 | 1.04 (1.00-1.08) | 0.034 | 1.03 (0.97-1.11) | 0.32 | 1.10 (0.95-1.27) | 0.20 | 0.99 (0.95-1.04) | 0.74 |
|  | Q5 | 1.04 (0.97-1.12) | 0.26 | 1.00 (0.96-1.05) | 0.91 | 1.12 (0.98-1.27) | 0.091 | 1.09 (0.95-1.25) | 0.20 | 1.06 (0.98-1.15) | 0.11 |
| *PMAD=Perinatal mood and anxiety disorders; MDP=Maternal mental disorders of pregnancy; SMI=Severe mental illness; SUI-T=Suicidal thoughts; SUB=Substance misuse; RR= Relative risk; CI=Confidence interval; ICE=Index of Concentration of Concentration at the Extremes; RUCA=Rural-Urban Commuting Area*  **Results for select outcomes excluded (NA) due to insufficient sample size*  **^a^***The p-values test the null hypothesis that heatwaves have no effect on maternal mental health outcomes for each strata (e.g., Age, Race)* | | | | | | | | | | | |

Supplemental Table 9. *p*-values for effect modification (*p*-EM) across subgroups during prolonged heatwave periods (lag0 to lag7)

|  | | **PMAD** | | **MDP** | | **SMI** | | **SUIT** | | **SUB** | |
| --- | --- | --- | --- | --- | --- | --- | --- | --- | --- | --- | --- |
| **Category** | **Subgroup** | **RR (95% CI^7^)** | **p-EM^a^** | **RR (95% CI)** | **p-EM^a^** | **RR (95% CI)** | **p-EM^a^** | **RR (95% CI)** | **p-EM^a^** | **RR (95% CI)** | **p-EM^a^** |
| Age | < 35 | 0.99 (0.97-1.01) | Ref. | 0.99 (0.97-1.01) | Ref. | 1.02 (0.98-1.05) | Ref. | 1.00 (0.97-1.03) | Ref. | 1.01 (0.99-1.03) | Ref. |
|  | >= 35 | 1.02 (0.98-1.05) | 0.12 | 1.01 (0.98-1.04) | 0.18 | 1.48 (1.34-1.63) | <0.0001 | 1.14 (1.08-1.22) | 0.00011 | 1.05 (1.00-1.09) | 0.17 |
| Race | White | 1.00 (0.97-1.02) | Ref. | 0.99 (0.97-1.01) | Ref. | 1.12 (1.06-1.17 | Ref. | 1.02 (0.99-1.06) | Ref. | 1.02 (1.00-1.04) | Ref. |
|  | Black | 0.97 (0.95-0.99) | 0.11 | 1.00 (0.98-1.02) | 0.034 | 1.06 (1.01-1.10) | 0.081 | 1.04 (1.00-1.09) | 0.44 | 1.00 (0.98-1.03) | 0.33 |
|  | Other Race | 1.05 (1.01-1.09) | 0.013 | 1.03 (0.99-1.08) | 0.19 | 1.05 (0.96-1.14) | 0.043 | 1.02 (0.97-1.07) | 0.91 | 1.10 (1.04-1.16) | 0.012 |
| Insurance | Private | 1.02 (0.99-1.05) | Ref. | 1.03 (1.00-1.06) | Ref. | 1.24 (1.14-1.35) | Ref. | 1.19 (1.12-1.25) | Ref. | 0.96 (0.93-1.00) | Ref. |
|  | Medicaid | 0.97 (0.94-0.99) | 0.0055 | 0.99 (0.97-1.01) | 0.044 | 1.05 (1.02-1.09) | 0.00030 | 1.02 (0.98-1.05) | <0.0001 | 1.03 (1.01-1.06) | 0.0015 |
|  | Self Pay | 1.05 (1.01-1.09) | 0.22 | 0.94 (0.90-0.98) | 0.24 | 0.99 (0.93-1.05) | <0.0001 | 0.92 (0.85-1.00) | <0.0001 | 1.01 (0.98-1.04) | 0.048 |
|  | Other | 1.11 (1.07-1.16) | 0.0015 | 0.99 (0.96-1.03) | <0.0002 | 0.90 (0.87-0.93) | <0.0001 | 1.03 (0.95-1.12) | 0.0059 | 1.10 (1.02-1.19) | 0.0016 |
| RUCA | Urban | 0.99 (0.96-1.01) | Ref. | 0.98 (0.96-1.00) | Ref. | 1.02 (0.98-1.06) | Ref. | 1.01 (0.96-1.06) | Ref. | 0.99 (0.97-1.02) | Ref. |
|  | Rural | 1.08 (0.94-1.23) | 0.20 | 1.00 (0.94-1.06) | 0.53 | NA | NA | 1.51 (1.17-1.96) | 0.0024 | 1.08 (1.01-1.14) | 0.016 |
|  | Suburban | 1.03 (0.98-1.07) | 0.10 | 1.03 (0.99-1.07) | 0.42 | 1.05 (0.98-1.13) | 0.021 | 1.08 (1.01-1.16) | 0.10 | 1.06 (1.01-1.11) | 0.015 |
| Region | Piedmont | 0.98 (0.95-1.01) | Ref. | 0.98 (0.95-1.00) | Ref. | 1.06 (1.01-1.12) | Ref. | 1.08 (1.00-1.18) | Ref. | 1.02 (0.99-1.05) | Ref. |
|  | Western | 1.09 (1.02-1.17) | 0.0039 | 0.98 (0.93-1.03) | 0.64 | 0.95 (0.86-1.06) | 0.023 | 1.09 (0.91-1.31) | 0.93 | 1.05 (1.00-1.11) | 0.34 |
|  | Coastal | 0.99 (0.96-1.01) | 0.0054 | 1.01 (0.98-1.03) | 0.10 | 1.06 (0.99-1.14) | 0.38 | 1.02 (0.98-1.06) | 0.70 | 1.00 (0.97-1.04) | 0.97 |
| ICE Race | Q5 | 1.08 (0.98-1.19) | Ref. | 1.04 (0.97-1.11) | Ref. | 1.13 (0.94-1.36 | Ref. | 1.79 (1.24-2.58) | Ref. | 1.06 (0.98-1.14) | Ref. |
|  | Q4 | 1.05 (0.98-1.12) | 0.63 | 1.35 (1.14-1.60) | 0.0048 | 1.35 (1.14-1.60) | 0.28 | 1.00 (0.87-1.15) | 0.0035 | 1.10 (1.01-1.18) | 0.57 |
|  | Q3 | 0.98 (0.93-1.03) | 0.081 | 1.14 (0.98-1.32) | 0.082 | 1.14 (0.98-1.32) | 0.26 | 1.08 (0.99-1.19) | 0.0094 | 1.02 (0.98-1.07) | 0.36 |
|  | Q2 | 1.02 (0.98-1.07) | 0.30 | 1.09 (0.98-1.20) | 0.22 | 1.09 (0.98-1.20) | 0.45 | 1.04 (0.94-1.14) | 0.0046 | 1.00 (0.96-1.04) | 0.17 |
|  | Q1 | 0.98 (0.94-1.02) | 0.067 | 1.00 (0.95-1.06) | 0.16 | 1.00 (0.95-1.06) | 0.46 | 1.09 (1.00-1.19) | 0.010 | 1.01 (0.98-1.05) | 0.26 |
| ICE Income | Q5 | 1.04 (0.97-1.12) | Ref. | 1.00 (0.96-1.05) | Ref. | 1.12 (0.98-1.27) | Ref. | 1.09 (0.95-1.25) | Ref. | 1.06 (0.98-1.15) | Ref. |
|  | Q4 | 1.03 (0.98-1.08) | 0.77 | 1.03 (0.97-1.11) | 0.21 | 1.03 (0.97-1.11) | 0.29 | 1.1 (0.95-1.27) | 0.95 | 0.99 (0.95-1.04) | 0.13 |
|  | Q3 | 0.97 (0.93-1.02) | 0.10 | 1.05 (0.95-1.14) | 0.25 | 1.05 (0.95-1.14) | 0.40 | 1.19 (1.02-1.38) | 0.43 | 1.03 (0.98-1.07) | 0.42 |
|  | Q2 | 1.00 (0.96-1.06) | 0.41 | 1.16 (1.06-1.27) | 0.91 | 1.16 (1.06-1.27) | 0.65 | 1.18 (1.07-1.30) | 0.36 | 1.05 (1.00-1.11) | 0.84 |
|  | Q1 | 1.00 (0.95-1.05) | 0.33 | 0.90 (0.84-0.97) | 0.23 | 0.9 (0.84-0.97) | 0.0044 | 0.95 (0.84-1.07) | 0.12 | 1.02 (0.97-1.07) | 0.34 |
| *PMAD=Perinatal mood and anxiety disorders; MDP=Maternal mental disorders of pregnancy; SMI=Severe mental illness; SUI-T=Suicidal thoughts; SUB=Substance misuse; RR= Relative risk; CI=Confidence interval; RUCA=Rural-Urban Commuting Area Codes; ICE=Index of Concentration of Extremes*  ^a^*p-EM= p-value used to assess effect modification (EM). Effect modification occurs when the relationship between the heatwaves and maternal health outcome varies across levels of a third variable (e.g., Age, Race, Region) and helps determine whether there is a significant difference in the effects between these groups. The statistic is calculated as: W = ((Beta_1 - Beta_2)^2) / (SE_Beta_1^2 + SE_Beta_2^2) Where: Beta_1 is the coefficient estimate for the first group (e.g., over age 35)), Beta_2 is the coefficient estimate for the reference group, SE_Beta_1 is the standard error of the coefficient for the first group, SE_Beta_2 is the standard error of the coefficient for the reference group.p-EM=p-value for Effect modification* | | | | | | | | | | | |

Supplemental Table 10. Cumulative relative risk (RR), 95% confidence interval (CI), and *p*-value estimates for maternal mental health conditions and long-term (lag0 to lag7) heatwave exposure periods stratified by heatwave intensity (low, moderate, and high).

| **Lag Period** | **Outcome** | **Low-Intensity RR (95% CI)** | **p** | **Moderate-Intensity RR (95% CI)** | **p** | **High-Intensity RR (95% CI)** | **p** |
| --- | --- | --- | --- | --- | --- | --- | --- |
| Lag0 to Lag3 | PMAD | 0.98 (0.93, 1.04) | 0.52 | 0.88 (0.78-0.99) | 0.57 | 1.01 (0.97-1.06) | 0.0013 |
|  | MDP | 0.98 (0.93, 1.03) | 0.46 | 1.17 (1.04-1.32) | 0.54 | 0.98 (0.93-1.04) | 0.99 |
|  | SMI | 1.11 (1.02, 1.20) | 0.011 | 1.56 (1.32-1.85) | 0.92 | 1.01 (0.90-1.12) | < 0.0001 |
|  | SUIT | 0.81 (0.74, 0.90) | < 0.0001 | 1.55 (1.30-1.86) | 0.30 | 0.97 (0.92-1.02) | 0.10 |
|  | SUB | 0.94 (0.89, 0.98) | 0.0073 | 1.10 (0.99-1.24) | 0.092 | 0.97 (0.93-1.01) | 0.56 |
| Lag0 to Lag7 | PMAD | 0.96 (0.93, 0.99) | 0.014 | 0.85 (0.77-0.93) | 0.98 | 1.00 (0.97-1.03) | 0.22 |
|  | MDP | 0.98 (0.95, 1.01) | 0.16 | 1.10 (1.01-1.20) | 0.10 | 1.03 (0.99-1.07) | 0.18 |
|  | SMI | 0.99 (0.94, 1.04) | 0.62 | 1.37 (1.19-1.58) | 0.00090 | 1.21 (1.08-1.35) | 0.072 |
|  | SUIT | 1.04 (0.98, 1.11) | 0.16 | 0.33 (0.28-0.38) | 0.83 | 0.99 (0.92-1.07) | 0.50 |
|  | SUB | 0.99 (0.97, 1.02) | 0.73 | 1.09 (1.00-1.18) | 0.14 | 1.02 (0.99-1.06) | 0.19 |
| *PMAD=Perinatal mood and anxiety disorders; MDP=Maternal mental disorders of pregnancy; SMI=Severe mental illness; SUI-T=Suicidal thoughts; SUB=Substance misuse; RR= Relative risk; CI=Confidence interval* | | | | | | | |

Supplemental Table 11. Cumulative relative risk (RR) estimates, 95% confidence intervals (CIs), and p-values for maternal mental health conditions for acute (lag0 to lag3) and prolonged (lag0 to lag7) heatwave exposure periods for alternative heatwave definitions (Heat Index and 3-Day 95%).

| **Lag Period** | **Outcome** | **3-Day 95% Heatwave RR (95% CI)** | **p** | **Heat Index RR (95% CI)** | **p** |
| --- | --- | --- | --- | --- | --- |
| Lag0 to Lag3 | PMAD | 1.03 (0.99-1.09) | 0.15 | 1.04 (0.93-1.18) | 0.48 |
|  | MDP | 1.01 (0.96-1.06) | 0.68 | 0.96 (0.87-1.07) | 0.48 |
|  | SMI | 1.08 (1.01-1.17) | 0.035 | 1.12 (0.94-1.34) | 0.19 |
|  | SUIT | 1.04 (0.96-1.12) | 0.38 | 0.82 (0.71-0.93) | 0.0026 |
|  | SUB | 1.03 (0.99-1.08) | 0.15 | 0.96 (0.86-1.07) | 0.43 |
| Lag0 to Lag7 | PMAD | 1.01 (0.98-1.03) | 0.48 | 1.04 (0.95-1.14) | 0.42 |
|  | MDP | 1.02 (1.00-1.05) | 0.10 | 0.87 (0.81-0.94) | 0.00040 |
|  | SMI | 1.02 (0.98-1.07) | 0.32 | 1.06 (0.94-1.20) | 0.35 |
|  | SUIT | 1.14 (1.08-1.21) | < 0.0001 | 1.21 (1.05-1.39) | 0.010 |
|  | SUB | 1.03 (1.00-1.05) | 0.040 | 0.94 (0.86-1.02) | 0.11 |
| *PMAD=Perinatal mood and anxiety disorders; MDP=Maternal mental disorders of pregnancy; SMI=Severe mental illness; SUI-T=Suicidal thoughts; SUB=Substance misuse; RR= Relative risk; CI=Confidence interval* | | | | | |

Supplemental Table 12. Cumulative relative risk (RR) estimates, 95% confidence intervals (CIs) and corresponding *p*-values for acute heatwave periods (lag0 to lag3) for maternal mental health outcomes for age categories 18 to 24, 25 to 29, 30 to 34, 35 to 39, and above 40 years old.

|  | **PMAD (n=392)** | | | **MDP (n=568)** | | | **SMI (n=64)** | | | **SUI-T (n=73)** | | | **SUB (n=365)** | | |
| --- | --- | --- | --- | --- | --- | --- | --- | --- | --- | --- | --- | --- | --- | --- | --- |
| **Age (Years)** | **n(%)** | **95% CI)** | **p^a^** | **n(%)** | **95% CI** | **p^a^** | **n(%)** | **95% CI** | **p^a^** | **n(%)** | **95% CI** | **p^a^** | **n(%)** | **95% CI** | **p^a^** |
| 18-24 | 180 (45.9) | 0.95 (0.92-0.99) | 0.012 | 247 (43.4) | 1.02 (0.98-1.07) | 0.23 | 25 (39.0) | 1.27 (1.17-1.39) | <0.0001 | 38 (52.0) | 1.09 (1.01-1.18) | 0.033 | 113 (30.9) | 1.01 (0.97-1.05) | 0.64 |
| 25-29 | 108 (27.5) | 0.94 (0.90-0.98) | 0.0073 | 175 (30.8) | 0.94 (0.90-0.98) | 0.0052 | 27 (42.1) | 1.09 (1.00-1.19) | 0.056 | 21 (42.4) | 1.22 (1.07-1.38) | 0.0023 | 131 (35.8) | 0.94 (0.91-0.98) | 0.0041 |
| 30-34* | 70 (17.8) | 1.02 (0.94-1.09) | 0.68 | 107 (18.8) | 1.15 (1.06-1.25) | 0.00050 | 8 (12.5) | NA | NA | 10 (13.6) | 1.43 (1.24-1.64) | < 0.0001 | 76 (20.8) | 1.17 (1.09-1.25) | < 0.0001 |
| 35-39* | 31 (7.9) | 0.98 (0.92-1.06) | 0.66 | 34 (5.9) | 1.02 (0.96-1.09) | 0.47 | 4 (6.2) | NA | NA | 3 (4.1) | NA | NA | 30 (8.2) | 1.07 (1.02-1.13) | 0.0083 |
| 40+* | 3 (0.7) | 1.02 (0.96-1.08) | 0.56 | 5 (0.8) | NA | NA | 0 (0.0) | NA | NA | 1 (1.3) | NA | NA | 15 (4.1) | 1.75 (1.39-2.20) | < 0.0001 |
| *PMAD=Perinatal mood and anxiety disorders; MDP=Maternal mental disorders of pregnancy; SMI=Severe mental illness; SUI-T=Suicidal thoughts; SUB=Substance misuse; RR= Relative risk; CI=Confidence interval*  **Results for select outcomes excluded (NA) due to insufficient sample size*  **^a^***TThe p-values test the null hypothesis that heatwaves have no effect on maternal mental health outcomes for each strata (e.g., 18-24)* | | | | | | | | | | | | | | | |

Supplemental Table 13. Cumulative relative risk (RR) estimates, 95% confidence intervals (CIs), and corresponding p-values for prolonged heatwave periods (lag0 to lag7) for maternal mental health outcomes for age categories 18 to 24, 25 to 29, 30 to 34, 35 to 39, and above 40 years old.

|  | **PMAD (n=773)** | | | **MDP (n=1106)** | | | **SMI (n=137)** | | | **SUI-T (n=119)** | | | **SUB (n=733)** | | |
| --- | --- | --- | --- | --- | --- | --- | --- | --- | --- | --- | --- | --- | --- | --- | --- |
| **Age (Years)** | **n(%)** | **95% CI** | **p^a^** | **n(%)** | **95% CI** | **p^a^** | **n(%)** | **95% CI** | **p^a^** | **n(%)** | **95% CI** | **p^a^** | **n(%)** | **95% CI** | **p^a^** |
| 18-24 | 322 (41.6) | 0.98 (0.95-1.00) | 0.061 | 457 (41.3) | 0.99 (0.97-1.01) | 0.42 | 57 (41.6) | 1.07 (0.99-1.15) | 0.10 | 55 (46.2) | 0.97 (0.92-1.02) | 0.29 | 215 (29.3) | 1.04 (1.01-1.07) | 0.010 |
| 25-29 | 225 (29.1) | 1.03 (1.00-1.06) | 0.051 | 356 (32.1) | 0.99 (0.97-1.02) | 0.65 | 57 (41.6) | 1.11 (1.05-1.16) | < 0.0001 | 37 (31.0) | 1.11 (1.06-1.17) | < 0.0001 | 272 (37.1) | 1.00 (0.97-1.02) | 0.71 |
| 30-34 | 141 (18.2) | 1.00 (0.96-1.03) | 0.79 | 199 (17.9) | 1.01 (0.98-1.04) | 0.69 | 11 (8.0) | 1.22 (1.14-1.31) | < 0.0001 | 19 (15.9) | 1.27 (1.18-1.38) | < 0.0001 | 168 (22.9) | 1.02 (0.99-1.05) | 0.12 |
| 35-39 | 67 (8.6) | 1.01 (0.97-1.05) | 0.57 | 89 (8.0) | 1.02 (0.99-1.05) | 0.13 | 12 (8.7) | 1.37 (1.26-1.49) | < 0.0001 | 5 (4.2) | 1.29 (1.20-1.37) | < 0.0001 | 56 (7.6) | 1.05 (1.00-1.10) | 0.045 |
| 40+* | 18 (2.3) | 1.26 (1.16-1.37) | < 0.0001 | 5 (0.4) | NA | NA | 0 (0.0) | NA | NA | 3 (2.5) | NA | NA | 22 (3.0) | 1.14 (1.08-1.21) | < 0.0001 |
| *PMAD=Perinatal mood and anxiety disorders; MDP=Maternal mental disorders of pregnancy; SMI=Severe mental illness; SUI-T=Suicidal thoughts; SUB=Substance misuse; RR= Relative risk; CI=Confidence interval*  **Results for select outcomes excluded (NA) due to insufficient sample size*  **^a^***The p-values test the null hypothesis that heatwaves have no effect on maternal mental health outcomes (e.g., PMAD, MDP) for each age category (e.g., 18-24)* | | | | | | | | | | | | | | | |

Supplemental Figure 1: Map of physiographic regions of North Carolina


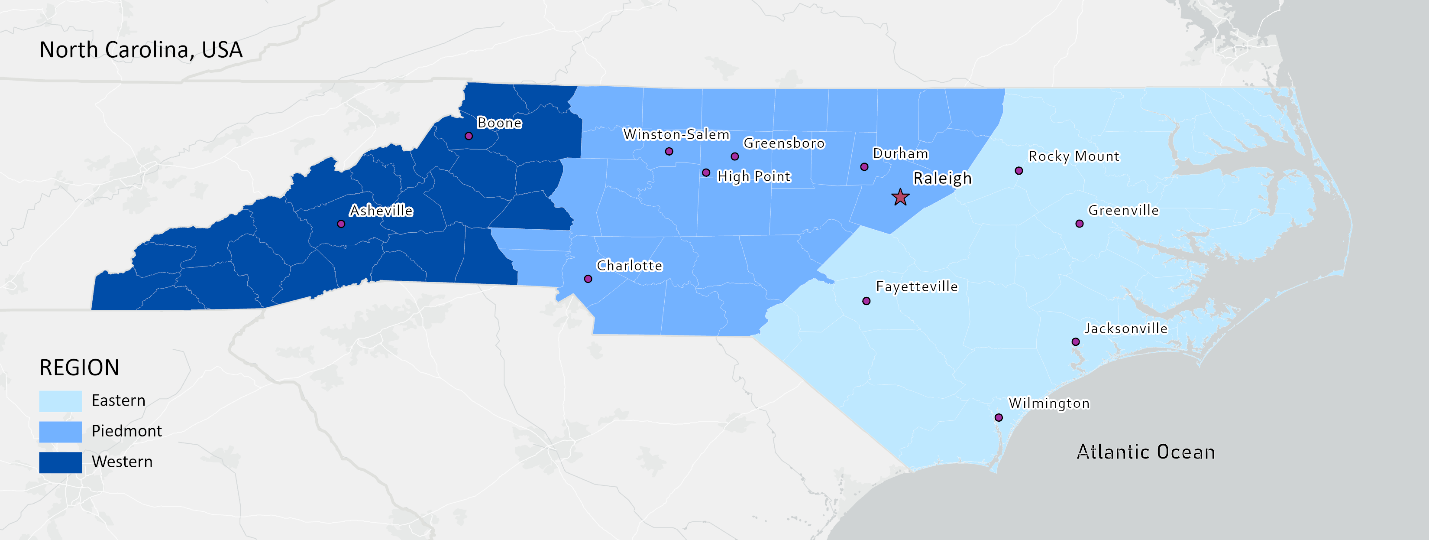


Supplemental Figure 2: Map of the distribution of Rural-Urban Commuting Area (RUCA) codes at the 2010 zip code tabulation area (ZCTA) level for North Carolina


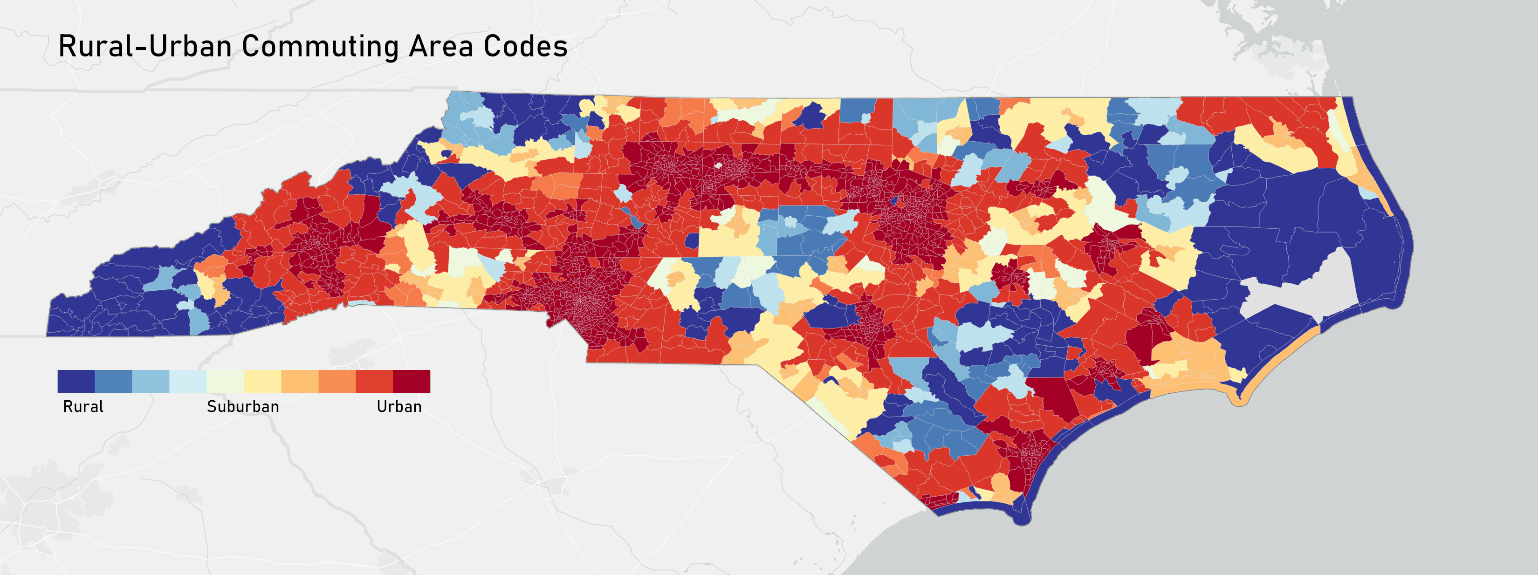


# Supplemental Figure 3: Index of Concentration at the Extremes (ICE) Income and ICE Race values by quantile at the zip-code tabulation area (ZCTA) level in North Carolina using data from the 2018 American Community Survey with 5-year estimates. For ICE Income, lower quantiles (e.g., Q1) represent primarily low-income neighborhoods, upper quantiles (e.g., Q5) represent high-income neighborhoods, and middle quantiles (Q2-Q4) represent mixed-income areas. For ICE Race, lower quantiles indicate majority non-white neighborhoods, upper quantiles indicate majority white neighborhoods and middle quantiles (Q2-Q4) represent mixed-race areas at the ZCTA level.


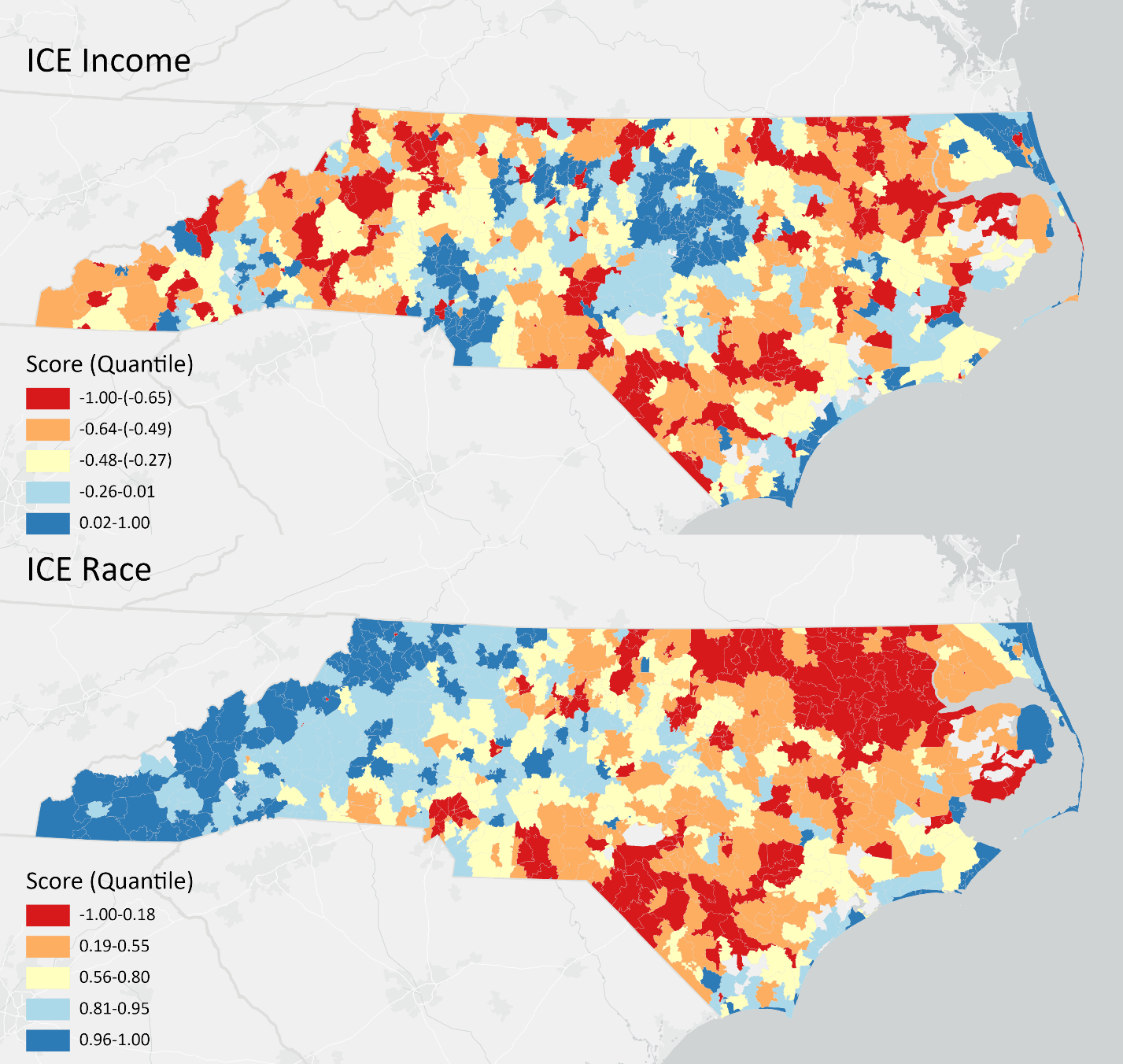


Supplemental Figure 4: Total number of excess heat factor (EHF) heatwave days, including those stratified by heatwave intensity for each zip code tabulation area (ZCTA) (n=804) from May to September 2011 to 2019.


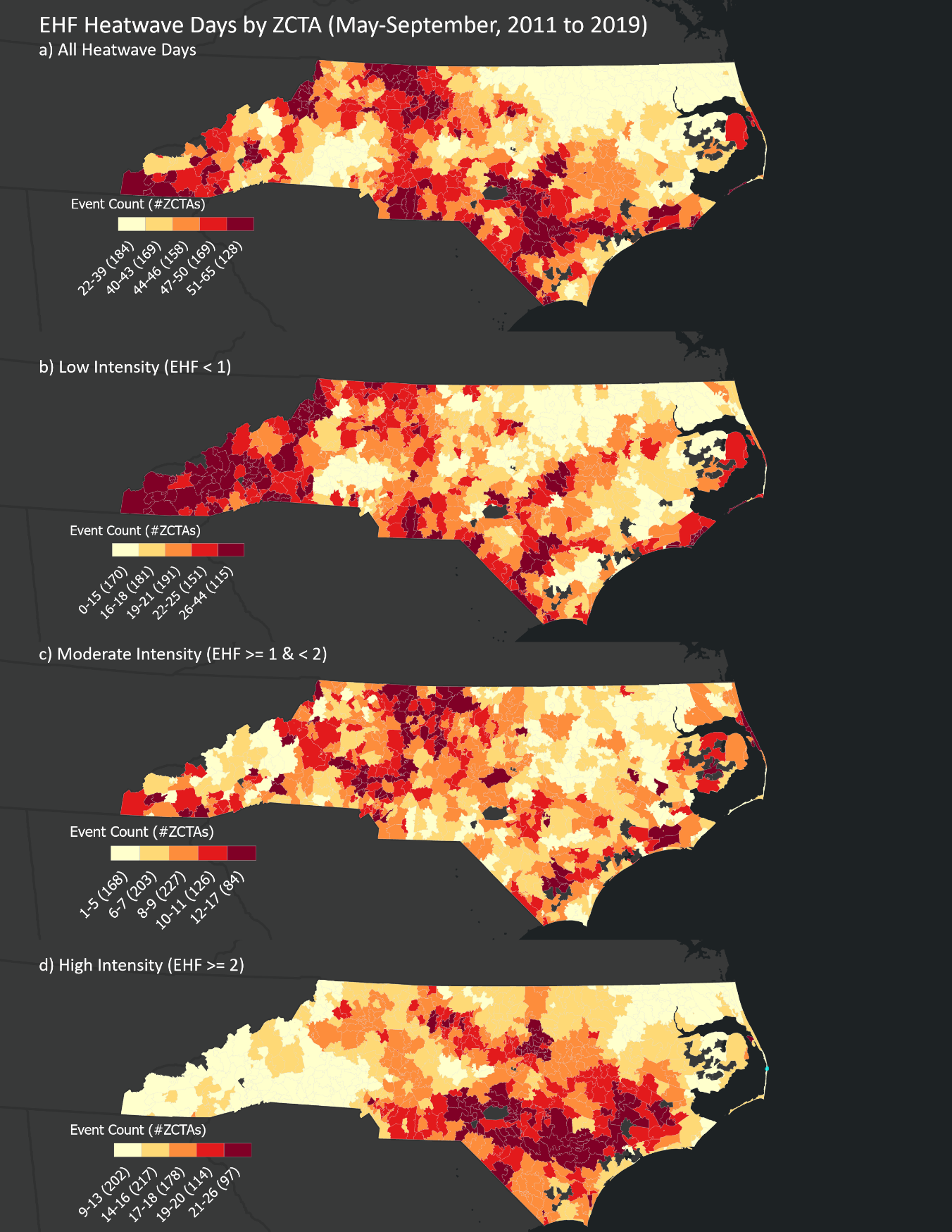


Supplemental Figure 5. Daily relative risk (RR) values and 95% confidence intervals (CIs) for heatwave days (lag0) and the seven consecutive days following (lag1 to lag7) for perinatal mood perinatal mood and anxiety disorders (PMAD), maternal mental disorders of pregnancy (MDP), severe mental illness (SMI), and substance abuse (SUB).


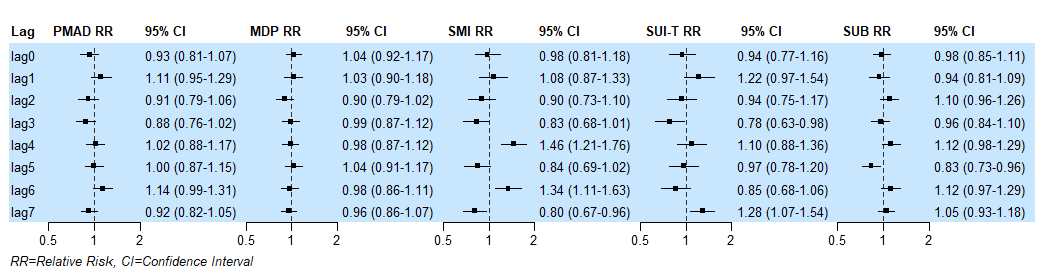


Supplemental Figure 6. Cumulative relative risk (RR) estimates for perinatal mood perinatal mood and anxiety disorders (PMAD), maternal mental disorders of pregnancy (MDP), severe mental illness (SMI), and substance abuse (SUB) for acute (lag0 to lag3) and long-term (lag0 to lag7) heatwave exposure periods stratified by heatwave intensity. Corresponding *p*-values are listed in Supplemental Table 6.


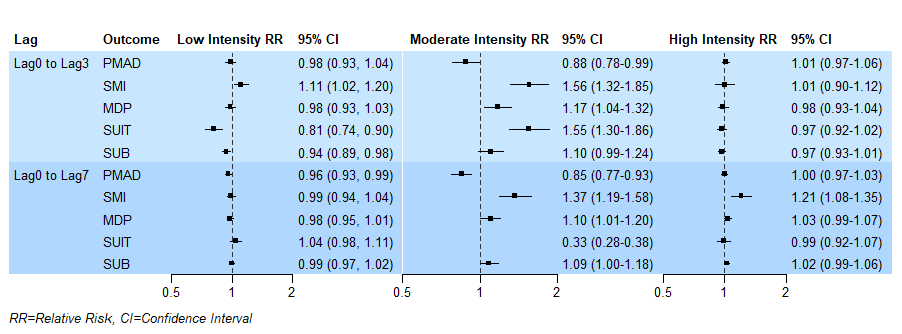


Supplemental Figure 7. Cumulative relative risk (RR) and 95% confidence interval (CI) estimates for perinatal mood perinatal mood and anxiety disorders (PMAD), maternal mental disorders of pregnancy (MDP), severe mental illness (SMI), and substance abuse (SUB) for acute (lag0 to lag3) and long-term (lag0 to lag7) heatwave exposure periods for alternative heatwave definitions (Heat Index and 3-Day 95%). Corresponding *p*-values are listed in Supplemental Table 7.


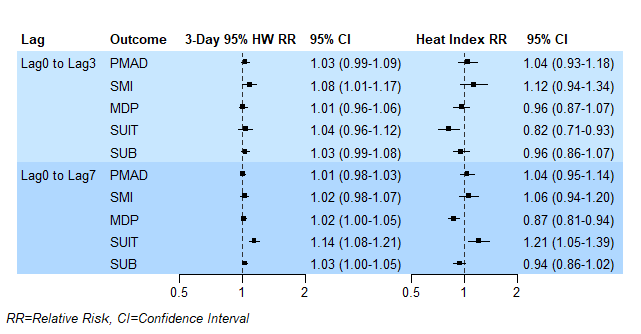


Supplemental Figure 8. Cumulative relative risk (RR) estimates and 95% confidence intervals (CIs) for acute heatwave periods (lag0 to lag3) for maternal mental health outcomes for age categories 18 to 24, 25 to 29, 30 to 34, 35 to 39, and above 40 years old. *p*-values for each estimate are listed in Supplemental Table 8.


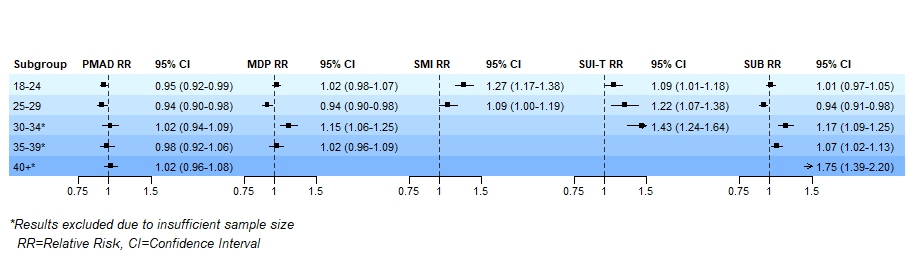


Supplemental Figure 9. Cumulative relative risk (RR) estimates and 95% confidence intervals (CIs) for prolonged heatwave periods (lag0 to lag7) for maternal mental health outcomes for age categories 18 to 24, 25 to 29, 30 to 34, 35 to 39, and above 40 years old. *p*-values for each estimate are listed in Supplemental Table 9.


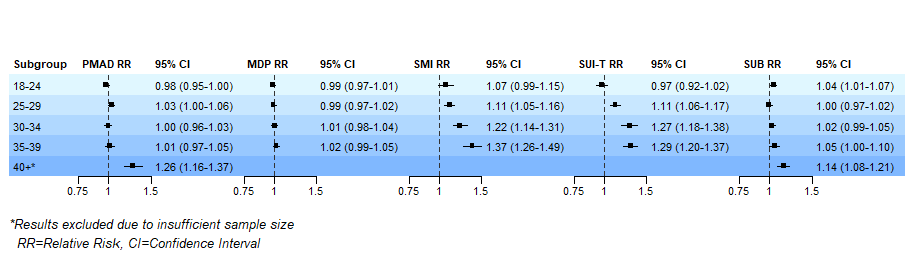

Supplement: Supplemental Figs. S1–S9 and Tables S1–S13 [file mmc1.docx]
